# Structure–Property Relationships in Hydrothermally Synthesized Monoclinic Scheelite BiVO_4_: Role of Preferential Crystallographic Orientation in Photocatalytic Activity

**DOI:** 10.3390/molecules31213804

**Published:** 2026-10-22

**Authors:** Freider Duran, Carlos Diaz-Uribe, William Vallejo, Ximena Vargas-Ramírez, Mario A. Millán-Franco, Edgar Mosquera-Vargas

**Affiliations:** 1 Grupo de Investigación en Fotoquímica y Fotobiología, Facultad de Ciencias Básicas, Universidad del Atlántico, Puerto Colombia 081007, Colombia; carlosdiaz@mail.uniatlantico.edu.co (C.D.-U.); williamvallejo@mail.uniatlantico.edu.co (W.V.); 2 Grupo de Procesos Químicos y Bioquímicos Sostenibles, Facultad de Ingeniería, Universidad del Atlántico, Puerto Colombia 081007, Colombia; ximenavargas@mail.uniatlantico.edu.co; 3 Centro de Estudios del Agua Universidad del Atlántico, Puerto Colombia 081007, Colombia; 4 Grupo de Transiciones de Fase y Materiales Funcionales, Facultad de Ciencias Naturales y Exactas, Universidad del Valle, Santiago de Cali 760042, Colombia; mario.millan@correounivalle.edu.co (M.A.M.-F.); edgar.mosquera@correounivalle.edu.co (E.M.-V.); 5 Centro de Excelencia en Nuevos Materiales, Universidad del Valle, Santiago de Cali 760042, Colombia

**Keywords:** monoclinic bismuth vanadate, BiVO_4_, photocatalysis, crystallographic orientation, charge transfer, methylene blue

## Abstract

Monoclinic scheelite BiVO_4_ is a visible-light photocatalyst whose performance is limited by charge–carrier recombination and inefficient charge transport. Here, a surfactant-free 3^2^ full factorial design was used to study the effects of initial pH (0.25, 0.50, and 0.75) and hydrothermal temperature (160, 180, and 200 °C) on the structural, morphological, optical, photoelectrochemical, and photocatalytic properties of BiVO_4_. All nine samples exhibited the monoclinic scheelite phase, with no detectable secondary crystalline phases within the XRD detection limits, and showed nearly invariant unit-cell parameters, V−O bond length, crystallite size, and optical band gap (2.44 ± 0.06 eV). In contrast, the I(040)/I(110) intensity ratio, used as a descriptor of preferential crystallographic orientation, varied from 2.32 to 3.36 and showed a strong correlation with the apparent rate constant for methylene blue (MB) discoloration (r = 0.931; Bonferroni-corrected *p* = 0.002), with the correlation remaining robust in leave-one-out sensitivity analysis. Higher ratios were associated with plate-like morphologies, lower charge-transfer resistance, and higher photocurrent. Response-surface analysis identified an optimum within the experimental domain, and the sample prepared at pH 0.50 and 180 °C reached 86.8% MB discoloration in 90 min (k_app_ = 0.0224 min^−1^), with an apparent rate constant approximately six times than of the least active sample. Scavenger tests indicated that O_2_ reduction pathways and photogenerated holes are the main contributors. These results identify preferential crystallographic orientation as a useful, though not exclusive, structural descriptor of photocatalytic activity in monoclinic BiVO_4_.

## 1. Introduction

Approximately 80% of the wastewater generated worldwide is estimated to be discharged into the environment without adequate treatment [1]. These wastewaters may contain a wide range of contaminants, including hydrocarbons, pesticides, pharmaceuticals, antibiotics, surfactants, synthetic dyes, and heavy metals. Many of these pollutants are highly ecotoxic, recalcitrant, and poorly biodegradable, which can promote their persistence and accumulation in aquatic ecosystems under continuous discharge [2,3,4]. This widespread contamination represents a major environmental and public health concern, emphasizing the need for treatment technologies capable not only of removing contaminants but also of transforming them into less harmful or environmentally benign products.

Conventional treatment technologies face significant limitations in dealing with these emerging and recalcitrant pollutants. Adsorption and coagulation are primarily separation-based processes that transfer contaminants from the aqueous phase to another phase rather than destroying them, whereas biological treatments can exhibit limited efficiency because certain contaminants can inhibit microbial activity or resist biodegradation [5,6]. Chemical oxidation processes, such as ozonation, can transform a broad range of contaminants; however, their degradation efficiency may depend strongly on the chemical structure of the target pollutant, and incomplete oxidation can result in the formation of transformation by-products. Consequently, there is a growing interest in degradation technologies capable of promoting extensive oxidation and, ideally, the mineralization of recalcitrant organic contaminants [7]. In this context, advanced oxidation processes (AOPs), particularly heterogeneous photocatalysis, have emerged as promising alternatives. These processes rely on the generation of highly reactive species, including hydroxyl radicals (∙OH), which can initiate a series of non-selective oxidation reactions leading to the degradation and, under suitable conditions, mineralization of persistent contaminants [8,9,10].

Heterogeneous photocatalysis employs semiconductor materials that are activated by absorbing photons with energies equal to or greater than their band-gap energy, generating electron–hole pairs. The subsequent migration of these photogenerated charge carriers to the semiconductor surface enables oxidation and reduction reactions involving adsorbed species, ultimately contributing to pollutant degradation [11]. Although this mechanism provides an opportunity to exploit solar radiation, widely investigated photocatalysts such as TiO_2_ and ZnO are predominantly activated by ultraviolet (UV) radiation, which represents only a limited portion of the solar spectrum. This limitation has stimulated the development of photocatalytic materials capable of operating under visible-light irradiation, including g-C_3_N_4_, CdS, WO_3_, Fe_2_O_3_, Ag_3_PO_4_, and BiVO_4_ [12,13,14].

Among these materials, BiVO_4_ has attracted considerable attention because of its suitable visible-light response, chemical stability, relatively low toxicity, and comparatively low cost. It is also one of the most widely studied photoanode materials for photoelectrochemical water splitting [15,16]. BiVO_4_ can crystallize in three polymorphic structures: monoclinic scheelite (ms), tetragonal zircon (tz), and tetragonal scheelite (ts). Among these polymorphs, monoclinic scheelite BiVO_4_ (ms-BiVO_4_) has been the most extensively investigated for visible-light photocatalysis. This interest is primarily associated with its relatively narrow band-gap energy (approximately 2.4 eV) and structural characteristics that favor visible-light absorption and can influence the separation and transport of photogenerated charge carriers [17,18,19]. Despite these advantages, the photocatalytic performance of BiVO_4_ remains constrained by intrinsic charge-transfer limitations, particularly the rapid recombination of photogenerated electron–hole pairs, relatively low charge-carrier mobility, and short hole-diffusion length [20]. These limitations reduce the number of photogenerated carriers that effectively reach the surface and participate in redox reactions, thereby restricting the overall photocatalytic efficiency.

Several strategies have therefore been investigated to improve the photocatalytic performance of BiVO_4_, including heterojunction formation, elemental doping, and deposition of metallic or other electron-accepting species [20,21,22,23,24,25]. Although these approaches can enhance charge separation and photocatalytic activity, they generally introduce additional components and may require multistep or comparatively complex synthesis procedures. An alternative strategy involves controlling crystal morphology and selectively exposing specific crystallographic planes, an approach commonly known as facet engineering [26]. Because different crystal facets can exhibit distinct surface energies, atomic arrangements, adsorption characteristics, and charge-transfer behavior, controlling their relative exposure provides a means of tailoring photocatalytic processes without necessarily introducing foreign phases or dopants. Conventional strategies for controlling facet development frequently employ directing agents, such as organic acids, polymers, or surfactants, which can remain adsorbed on the particle surface or require additional post-treatment steps for their removal [27,28,29].

Among the available methods for synthesizing BiVO_4_, hydrothermal processing is particularly attractive because it enables controlled crystal nucleation and growth under relatively moderate conditions and can promote morphological evolution through dissolution-recrystallization processes without necessarily requiring external directing agents [30,31,32]. Nevertheless, the structural, morphological, and optical properties of the resulting semiconductors are strongly dependent on the hydrothermal synthesis parameters. In particular, the pH of the reaction medium can modify precursor speciation, solubility, nucleation behavior, and phase stability, whereas the hydrothermal temperature directly affects crystal growth kinetics, crystallinity, particle morphology, and defect formation. Previous studies have extensively examined the effects of individual synthesis parameters; however, the coupled influence of pH and hydrothermal temperature on the structural and electronic characteristics of BiVO_4_ remains comparatively underexplored [33,34,35,36,37]. Evaluating these parameters independently may overlook interaction effects arising from their simultaneous variation, potentially obscuring the conditions required to achieve a favorable combination of crystallinity, morphology, optical response, and photocatalytic performance.

In this work, a surfactant-free 3^2^ full factorial design was used to evaluate the combined effects of initial pH and hydrothermal temperature on monoclinic scheelite BiVO_4_. Rather than introducing dopants, co-catalysts, or structure-directing agents, we sought to identify which structural features are sensitive to these synthesis variables and how they are associated with charge-transfer behavior and visible-light photocatalytic activity. The I(040)/I(110) diffraction intensity ratio was used as a descriptor of preferential crystallographic orientation and was examined in relation to morphology, Mott–Schottky, EIS, and photocurrent data, as well as methylene blue (MB) discoloration kinetics, using factorial and correlation analysis. MB was employed as a comparative probe to rank photocatalytic performance under identical conditions, rather than to evaluate mineralization.

## 2. Results and Discussion

### 2.1. Structural Study

The crystalline phases of the synthesized materials were investigated by XRD. Figure 1a shows the XRD patterns obtained for the nine BiVO_4_ samples synthesized under different hydrothermal temperature conditions. The diffraction peaks observed at 2θ values of approximately 18.7°, 19.0°, 28.9°, 30.6°, 34.6°, 35.3°, 40°, 42.3°, 46.8°, 47.3°, 50.21°, 53.2°, 58.3° and 59.6° were assigned to the (110), (011), (121)/(−121), (040), (200), (002), (211), (051), (240), (042), (202), (161), (−321) and (321) crystallographic planes, respectively. All these reflections are consistent with the monoclinic scheelite structure of BiVO_4_ (ms-BiVO_4_), according to ICDD PDF 00-014-0688 [38,39]. Furthermore, the characteristic splitting of the (200) and (002) reflections at approximately 34.6° and 35.3°, respectively, provides additional evidence for the monoclinic scheelite phase and differentiates it from the tetragonal scheelite structure. The absence of a prominent reflection near 2θ = 24.4° also indicates that no detectable tetragonal zircon BiVO_4_ phase is present within the detection limits of the XRD analysis.

The formation of BiVO_4_ can be described through a pH-dependent sequence involving precursor hydrolysis, condensation, and subsequent interaction between bismuth- and vanadium-containing species, consistent with mechanisms previously proposed for hydrothermal BiVO_4_ synthesis [40,41]. In the first stage, the Bi^3+^ may undergo partial hydrolysis according to Equation (1), followed by interaction with nitrate-containing species to generate a bismuth oxynitrate intermediate, as represented in Equation (2). This intermediate can subsequently react with vanadate species, resulting in BiVO_4_ precipitation according to Equation (3).

*Bi*^3+^ + *H*_2_O ⇌ *Bi*(*OH*)^2+^ + *H*^+^ (1)

(2) $${\mathrm{NO}}_{3}^{-}+{\mathrm{Bi}\left(\mathrm{OH}\right)}^{2+}\to {\mathrm{BiONO}}_{3}↓+H^{+}$$

(3) $${\mathrm{BiONO}}_{3}+{\mathrm{VO}}_{3}^{-}\to {\mathrm{BiVO}}_{4}\left(S\right)+{\mathrm{NO}}_{3}^{-}$$

It has been proposed that BiONO_3_ can act as an intermediate toward the formation of tetragonal zircon BiVO_4_, whose formation is kinetically favored under appropriate conditions [42,43]. This phase-selection behavior is particularly relevant when comparing the present results with previous hydrothermal syntheses because the crystal structure of BiVO_4_ has been shown to depend strongly on synthesis conditions such as pH and temperature. For example, Lenczewska et al. reported that hydrothermal processing at pH 7.5 and 200 °C could produce tetragonal zircon-type BiVO_4_, whereas monoclinic material was obtained after subsequent thermal treatment [44]. In contrast, all nine materials synthesized in the present work directly exhibit the monoclinic scheelite phase, despite the systematic variation in pH and hydrothermal temperature.

The difference can be associated with the strong acidic synthesis environment employed here. Although the precursors are initially dissolved in 4 M HNO_3_, the medium remains strongly acidic after pH adjustment (pH 0.25–0.75), and the hydrolysis equilibrium represented by Equation (1) is therefore expected to favor Bi^3+^ over hydrolyzed bismuth species. This condition may restrict the formation of BiONO_3_ and consequently suppress the pathway associated with the tetragonal zircon phase. Thus, the high acidity used in the present synthesis appears to provide a relatively robust chemical environment for the direct formation of monoclinic scheelite BiVO_4_ across the investigated synthesis domain. This interpretation is consistent with the XRD results, and the Raman analysis presented below provides complementary evidence for the same structural assignment.

Although no detectable phase transformation was observed, systematic differences in the relative intensities of selected diffraction reflections were evident among the nine samples. This observation is important because changes in relative peak intensity can arise from preferential crystallographic orientation even when the phase composition remains unchanged. Therefore, structural analysis was extended beyond phase identification by calculating descriptors derived from the XRD patterns and Raman spectra, including V–O bond length, preferential crystallographic orientation, crystallite size, anisotropy, and unit-cell volume.

The V–O bond length was estimated using the empirical Hardcastle–Wachs correlation based on the position of the symmetric νs(V–O) stretching mode in the Raman spectrum [45]. The calculated V–O bond lengths were approximately 1.69 Å, with only minor variations among the samples, on the order of 0.002 Å (Table 1).

Such small variations indicate that the local V–O bonding environment remained essentially stable throughout the investigated synthesis domain. Regarding preferential crystallographic orientation, previous studies have associated the {010} and {110} facets of monoclinic scheelite BiVO_4_ with different surface redox processes, with the {010} facet commonly associated with reduction reactions and the {110} facet with oxidation reactions [40,46,47,48]. Accordingly, the relative contribution of the {010} family was evaluated using the intensity ratio I(040)/I(110), obtained from the pseudo-Voigt fitting of the corresponding diffraction reflections.

The I(040)/I(110) structural descriptor ranged from 2.32 to 3.36 among the nine samples (Table 1). For a randomly oriented powder, the I(040)/I(110) ratio calculated from the crystal structure of monoclinic scheelite BiVO_4_ using Cu K_α1_ and integrated peak intensities is 0.84 [49]. The measured values therefore exceed the reference value by a factor of 2.8–4.0, indicating a pronounced preferential crystallographic orientation associated with the (040) reflection and the [010] direction. This variation is considerably larger than that observed for the V–O bond length and other bulk structural descriptors, suggesting that preferential orientation is more sensitive to the synthesis conditions than the local crystal structure. The contributions of the (040) and (110) reflections to the observed variation were quantified through variance decomposition on a logarithmic scale. For the nine samples, variations in I(040) accounted for approximately 80% of the total variance, whereas I(110) contributed approximately 20%. Thus, the changes in the I(040)/I(110) ratio were dominated primarily by changes in the intensity of the (040) reflection, while the contribution of the (110) reflection remained comparatively stable. This result indicates that the synthesis conditions predominantly modified the relative development of the {010}-related crystallographic orientation rather than producing comparable changes in the {110} contribution.

The average crystallite sizes, calculated using the Scherrer equation, ranged from 57 to 65 nm. These values are smaller than those reported in some hydrothermal BiVO_4_ systems. For example, Lenczewska et al. reported crystallite sizes of approximately 84 nm for as-synthesized monoclinic BiVO_4_ and 119 nm for their as-synthesized tetragonal BiVO_4_ material [44]. This difference should not be attributed solely to hydrothermal temperature because the synthesis routes differ in precursor chemistry, pH, microwave-assisted versus conventional hydrothermal processing, dopant incorporation, and subsequent thermal treatment. Nevertheless, the comparatively smaller crystallite dimensions obtained here indicate that the selected synthesis conditions favor the formation of nanoscale coherent domains without requiring a post-synthesis calcination step.

The crystallite-size behavior also differs from systems in which increasing temperature produces pronounced crystal coarsening. In the comparison study, crystallite growth occurred after annealing and was attributed to Ostwald ripening. In the present study, by contrast, the crystallite sizes remained within the relatively narrow range of 57–65 nm throughout the synthesis matrix, suggesting that the investigated pH-temperature combinations primarily affected crystal development and orientation rather than producing extensive changes in coherent domain size.

The anisotropy, quantified by the D(040)/D(121) ratio, remained between 1.2 and 1.3 for all samples, indicating that the crystallite domains were approximately 20–30% larger along the [010] direction than along the direction associated with the (121) planes. Furthermore, the unit-cell parameters obtained from indexing the XRD patterns showed only minor variations among the nine samples, remaining within the range reported for monoclinic scheelite BiVO_4_ [50,51]. The simultaneous stability of the unit-cell parameters and V–O bond length further indicates that the synthesis variables did not induce substantial lattice distortion or compositional changes within the BiVO_4_ structure.

These results indicate that the synthesis conditions had a relatively limited effect on the bulk crystallographic characteristics of BiVO_4_. The crystalline phase, V–O bond length, crystallite size, anisotropy, and unit-cell parameters remained comparatively stable across the experimental domain, whereas the I(040)/I(110) ratio exhibited a substantially greater variation. This behavior suggests that the principal structural consequence of changing pH and hydrothermal temperature was not phase transformation or significant modification of the bulk lattice but rather the preferential development of particular crystallographic orientations. Such decoupling is important for photocatalytic materials because surface orientation can be modified without necessarily altering the fundamental crystal structure.

This behavior can be compared with previous hydrothermal studies in which pH variations modified the morphology and relative facet development of BiVO_4_ while maintaining the monoclinic scheelite structure. The literature therefore supports the interpretation that surface development can respond more strongly to synthesis conditions than the underlying crystal lattice. In contrast, the comparison study demonstrates that sufficiently different synthesis and thermal-treatment conditions can also induce phase changes and substantial crystallite growth. The present results therefore indicate that the strongly acidic precursor environment, combined with controlled hydrothermal treatment, provides a synthesis window in which phase stability is maintained while preferential orientation can be tuned.

Figure 1b shows the I(040)/I(110) preferential orientation descriptor as a function of reaction pH and hydrothermal treatment temperature. The data reveal a non-monotonic dependence on the synthesis variables, with the highest degree of preferential orientation toward the [010] related direction observed for the sample synthesized at pH 0.50 and 180 °C. The descriptor decreased at both lower and higher hydrothermal temperatures, indicating that increasing temperature does not produce a simple progressive enhancement of preferential orientation.

This behavior can be interpreted in terms of the competition among nucleation, crystal growth, and dissolution processes during hydrothermal treatment. Increasing the hydrothermal temperature generally accelerates the kinetics of crystal growth; however, the relative growth rates of different crystallographic directions are not necessarily equivalent. Because the relative growth of the [010] direction can be slower than that of other crystallographic directions, an intermediate temperature may favor the development of crystals with a greater contribution from the {010} orientation. At still higher temperatures, enhanced dissolution and changes in nucleation density may modify this balance, limiting the development of a strongly preferred orientation [30,40].

The maximum I(040)/I(110) ratio at 180 °C therefore suggests that this temperature provides a favorable kinetic balance between nucleation, anisotropic growth, and dissolution under the investigated pH conditions. The subsequent decrease at 200 °C indicates that excessive thermal acceleration does not necessarily increase the preferential development of the {010} orientation. This interpretation is consistent with the broader observation that hydrothermal temperature can influence crystallite development and morphology through competing growth and dissolution processes. Importantly, however, the present results demonstrate that these kinetic changes can occur without inducing a detectable phase transformation.

In addition, this behavior is consistent with previous observations for hydrothermally synthesized monoclinic BiVO_4_, where changes in pH produced modifications in crystal thickness and relative facet development while preserving the monoclinic scheelite phase over the investigated conditions [30,52]. Similarly, theoretical studies have shown that the different surfaces of monoclinic BiVO_4_ possess distinct surface energies, providing a thermodynamic basis for the preferential development of crystallographic facets under suitable synthesis conditions [53]. Consequently, changes in the chemical environment that modify precursor speciation, supersaturation, nucleation rate, or crystal-growth kinetics can alter the relative development of crystal facets without necessarily producing measurable changes in the bulk crystal structure.

Finally, the I(040)/I(110) ratio should be interpreted as a semiquantitative descriptor of preferential crystallographic orientation, not as a direct measurement of the exposed areas of the {010} and {110} facets. Nevertheless, its variation across the synthesis matrix provides a useful structural parameter for evaluating how pH and hydrothermal temperature affect the anisotropic development of BiVO_4_ crystal. The relevance of this descriptor to photocatalytic performance is examined in the following sections by correlating preferential crystallographic orientation with the optical, electrochemical, and photocatalytic properties of the synthesized BiVO_4_ materials.

### 2.2. Morphological Analysis

The morphology of the nine materials was examined by SEM (Figure 2). All samples consisted of aggregates of submicrometer to micrometer sized primary particles, with the aggregates displaying a polycrystalline character rather than well-defined single-crystal morphologies. This morphological evolution contrasts with the relatively small variations observed in the bulk structural parameters obtained from XRD, including crystallite size, unit-cell dimensions, and V–O bond length, indicating that the synthesis conditions primarily affected crystal habit and particle assembly rather than the BiVO_4_ crystal structure itself. The habit of the primary particles varied systematically across the synthesis domain, with a non-monotonic dependence on hydrothermal temperature that is consistent with the preferential-orientation trends identified by XRD.

The materials treated at 160 °C exhibited primary particles with poorly developed crystal habits. The sample obtained at pH 0.25 (M1) consisted of loosely branched, dendrite-like aggregates assembled from fine equiaxed particles of approximately 0.2–0.5 μm, with no discernible flat faces. At pH 0.50 and 0.75 (M2 and M3), the particles were larger, approximately 0.5–1.5 μm, but appeared rounded and extensively coalesced, with smooth necks between neighboring particles and rounded rather than polyhedral outlines. These features suggest that crystal growth under these conditions did not result in the development of well-defined low-index facets. However, because SEM provides morphological rather than mechanistic information, the observed rounded morphology should not be attributed exclusively to a particular nucleation or dissolution pathway without complementary kinetic evidence.

At 180 °C, the particle habit changed markedly and became dependent on the initial pH. The sample synthesized at pH 0.25 (M4) still consisted of rounded nodular particles of approximately 0.4–1.0 μm, with only incipient faceting. In contrast, the samples obtained at pH 0.50 (M5) and pH 0.75 (M6) were composed of well-defined plate-like crystals. In M5, the plates displayed lateral dimensions of approximately 0.8–1.5 μm and an apparent thickness of 0.15–0.3 μm when observed edge-on. The plates in M6 were morphologically similar but somewhat smaller in lateral dimension and tended to assemble into flower-like aggregates. The emergence of plate-like morphologies at these intermediate-temperature conditions agrees with the XRD results, for which M5 and M6 exhibited the highest I(040)/I(110) ratios (3.36 and 2.98, respectively). This correlation suggests that the development of the plate-like habit is associated with a stronger preferential contribution of crystallographic orientations related to the {010} family.

For monoclinic scheelite BiVO_4_, anisotropic crystal growth can produce plate-like morphologies associated with preferential development of specific crystallographic faces. Nevertheless, the assignment of the observed plate surfaces specifically to {010} should be regarded as a structural interpretation based on the combined XRD-SEM evidence rather than as a direct facet identification, because conventional SEM does not provide crystallographic indexing of individual faces. In addition, plate-like crystals tend to lie flat on the sample holder in Bragg–Brentano geometry, which enhances the (0k0) reflections. The high I(040)/I(110) ratios observed for M5 and M6 are therefore consistent with plate-like morphology in which the broad faces are normal to the [010] direction. Thus, the agreement between the increased I(040)/I(110) ratio and the appearance of plate-like particles provides morphological support for the preferential-orientation analysis, but not a direct quantitative determination of exposed {010} surface area.

At 200 °C, the plate-like habit was no longer observed. The sample synthesized at pH 0.50 (M8) consisted of angular, polyhedral crystallites of approximately 0.5–1.2 μm, with flat faces and sharp edges but a roughly equiaxed outline, whereas the sample obtained at pH 0.75 (M9) presented a dense aggregate of smaller angular particles of approximately 0.3–0.8 μm. Both samples were therefore more clearly faceted than the materials synthesized at 160 °C, but their morphologies were less anisotropic than those observed for M5 and M6. The sample synthesized at pH 0.25 (M7) formed dense aggregates of fine particles whose individual habit could not be resolved at the magnification employed.

The transition from anisotropic plates at 180 °C to more equiaxed polyhedral particles at 200 °C is consistent with the non-monotonic variation in the I(040)/I(110) ratio and suggests that increasing the hydrothermal temperature beyond the optimum does not necessarily enhance anisotropic crystal development. Rather than assigning this transition solely to accelerated dissolution and secondary nucleation, the SEM and XRD results support a broader interpretation in which temperature modifies the relative rates of nucleation, crystal growth, aggregation, and possible dissolution-reprecipitation processes. This interpretation is also consistent with the relatively stable crystallite sizes and unit-cell parameters obtained by XRD despite the pronounced changes in micrometre-scale particle habit, indicating that the synthesis conditions affect the organization and growth of crystallites into larger aggregates without substantially altering the underlying monoclinic BiVO_4_ structure.

The combined XRD and SEM results therefore establish a clear processing–structure–morphology relationship. The strongly acidic synthesis environment produced single-phase monoclinic scheelite BiVO_4_ throughout the experimental domain, while changes in pH and hydrothermal temperature primarily modified crystallographic orientation and particle habit. In particular, the highest I(040)/I(110) ratios occurred under the conditions that produced the most pronounced plate-like morphologies. This correspondence strengthens the interpretation of the diffraction ratio as a descriptor of preferential crystallographic orientation and indicates that pH and temperature act mainly by controlling anisotropic crystal development rather than phase formation in the present synthesis window.

Ranking the samples according to the I(040)/I(110) descriptor also reveals a qualitative correspondence with their SEM morphologies: the two samples with the highest values, M5 and M6, displayed plate-like crystals; samples with lower but still substantial ratios, particularly those synthesized at 200 °C, exhibited angular and polyhedral crystallites; whereas the lowest values were associated with dendritic, rounded, or poorly developed morphologies.

### 2.3. Vibrational Spectroscopy

The FTIR-ATR spectra of the nine samples (Figure 3a) displayed vibrational features characteristic of the BiVO_4_ structure, in agreement with previously reported results [54,55]. Three main groups of absorption bands were identified in the 900–400 cm^−1^ region, corresponding predominantly to vibrational modes associated with the VO_4_^3–^ tetrahedral units and Bi–O bonds. The band observed in the 738–836 cm^−1^ range was assigned to the *ν*(V–O) stretching vibration, whereas the bands detected between 518 and 678 cm^−1^ were associated with Bi–O-related vibrational modes and deformation vibrations of the VO_4_^3−^ units. Finally, the absorption band located in the 461–506 cm^−1^ range was attributed to the symmetric deformation mode, δ_s_(VO_4_^3−^) [54,55].

The relatively broad ranges observed for these vibrational bands among the nine samples indicate that the local bonding environment of BiVO_4_ exhibits subtle variations as a function of the synthesis conditions. Variations in reaction pH and hydrothermal temperature can modify precursor speciation, nucleation, crystal-growth kinetics, and the local structural organization, which may consequently influence the position, width, and relative intensity of the Bi–O and V–O vibrational bands. However, because the fundamental FTIR band pattern remains essentially unchanged across all nine samples, these variations should be interpreted as local rather than as evidence of a substantial modification of the BiVO_4_ crystal structure. The absence of additional well-defined bands attributable to secondary vanadium- or bismuth-containing phases is consistent with the phase-purity results obtained by XRD. Therefore, FTIR provides complementary information on the local bonding environment, whereas phase identification is primarily established from the XRD analysis discussed above.

The Raman spectra (Figure 3b) exhibited a set of prominent vibrational modes characteristic of monoclinic scheelite BiVO_4_ in all nine samples. The spectra were dominated by an intense band at approximately 827 (A_g_) cm^−1^, accompanied by a weaker band near 712 (A_g_) cm^−1^. These bands are assigned to the symmetric (ν_s_) and asymmetric (ν_as_) stretching modes of the V–O bonds in the VO_4_^3−^ tetrahedral units, respectively. The bands located at approximately 367 (A_g_) and 328 (A_g_) cm^−1^ were attributed to symmetric (δ_s_) and asymmetric (δ_as_) O–V–O deformation modes of the VO_4_^3−^ tetrahedra, respectively, whereas the signals observed at approximately 212 (A_g_) and 129 (A_g_) cm^−1^ were assigned to external lattice modes involving translational and librational motions of the VO_4_^3−^ groups and Bi^3+^ ions [41,56,57]. The positions of the principal Raman bands are in close agreement with those reported for monoclinic BiVO_4_, for which bands near 830, 709, 369, 328, 213, and 129 cm^−1^ have been reported.

The Raman results provide independent spectroscopic support for the phase assignment obtained by XRD. In particular, the strong V–O stretching mode at ~ 827 cm^−1^ and the accompanying band at ~712 cm^−1^ closely match the characteristic Raman response reported for monoclinic BiVO_4_, while the absence of the bands characteristic of the tetragonal zircon-type phase further supports the XRD conclusion that all nine materials crystallized predominantly as monoclinic BiVO_4_. In the study by Lenczewska et al., the monoclinic phase exhibited V–O stretching bands at 830 and 709 cm^−1^, whereas the tetragonal zircon-type phase displayed a substantially different Raman pattern, including a high-frequency V–O stretching band near 854 (A_1g_) cm^−1^ [44]. The close correspondence of the present spectra with the former pattern is therefore consistent with the assignment of all nine samples to the monoclinic phase.

The Raman spectra also complemented the FTIR-ATR results. The intense Raman band at ~827 cm^−1^ falls within the V–O stretching region identified by FTIR-ATR (738–836 cm^−1^), while the Raman deformation modes between approximately 324 and 368 cm^−1^ provide complementary information on the vibrational behavior of the VO_4_^3−^ tetrahedra. The agreement between the two spectroscopic techniques indicates that the fundamental V–O bonding framework is preserved throughout the synthesis domain, despite the changes in particle morphology observed by SEM and the variations in preferential orientation detected by XRD. Thus, the pH and hydrothermal temperature conditions modify crystal development and particle habit without producing a detectable change in the fundamental vibrational fingerprint of monoclinic BiVO_4_.

### 2.4. Optical and Electrochemical Characterization

The diffuse reflectance spectra (Figure 4a) of all synthesized samples exhibited similar, well-defined absorption edges in the range of 508–520 nm, consistent with the values reported for monoclinic BiVO_4_ (approximately 500–510 nm) [58,59,60]. The optical band-gap energy was estimated from Tauc plots constructed using the Kubelka–Munk function derived from the diffuse reflectance data, using the relationship (F(R)*hν*)^2^ versus *hν* for an allowed direct transition, as reported for crystalline BiVO_4_. The optical band-gap energy remained essentially constant across the synthesis domain, with an average value of 2.44 eV and a standard deviation of ±0.06 eV (Figure 4b). The small variation in *E_g_*, together with the absence of a systematic shift in the absorption edge, indicates that changes in pH and hydrothermal temperature did not substantially modify the electronic band structure of the BiVO_4_ lattice. This result is consistent with the XRD and Raman analyses, which showed preservation of the monoclinic scheelite structure across all nine samples.

The photoluminescence spectra (Figure 4c) of all synthesized samples exhibited a single broad emission band extending from approximately 490 to 600 nm, with the maximum centered at 544 nm (2.28 eV), in agreement with values reported for monoclinic BiVO_4_ [61]. The emission maximum was essentially invariant across the synthesis domain, further supporting the stability of the fundamental electronic structure inferred from the diffuse-reflectance measurements. The emission energy was approximately 0.17 eV lower than the optical band-gap energy determined from the Tauc analysis, indicating a Stokes shift that may be associated with radiative recombination involving localized or relaxed electronic states rather than a purely band-to-band transition [62,63]. Because the PL spectra were not deconvoluted into distinct defect-related contributions, the precise origin of this emission cannot be assigned exclusively to a particular defect or localized state.

The integrated emission intensity varied by a factor of 2.8 across the series, with the samples treated at 180 °C displaying the lowest values and those obtained at 160 °C the highest. Since the spectra were acquired under identical instrumental conditions but without normalization to sample mass or absorbed photon flux, these intensities are compared qualitatively and are not interpreted here as a quantitative measure of the recombination rate. Nevertheless, the lower PL intensity observed for the 180 °C samples is qualitatively consistent with the electrochemical results presented below, which indicate more favorable interfacial charge-transfer behavior under these synthesis conditions. The agreement between the two measurements suggests that the 180 °C materials may exhibit more efficient separation or extraction of photogenerated charge carriers, although PL intensity alone cannot distinguish between reduced radiative recombination and changes in absorption, collection efficiency, or nonradiative pathways.

The Mott–Schottky plots are shown in Figure 5a, and the electrochemical parameters are listed in Table 2. All samples exhibited positive slopes in the linear region, consistent with *n*-type semiconductor behavior, in agreement with previous reports for monoclinic BiVO_4_ [64,65]. The flat-band potential was converted to the reversible hydrogen electrode (RHE) scale according to E(RHE) = E(SCE) + 0.2412 + 0.0592 (pH), where E(RHE) is the potential converted to the reversible hydrogen electrode scale, E(SCE) is the potential measured against a saturated calomel electrode (SCE), 0.2412 V is the standard potential of the *SCE* relative to the standard hydrogen electrode at 25 °C, 0.0592 V is the Nernstian slope per pH unit at 25 °C (2.303 RT/F) and pH is the pH of the electrolyte solution used [66]. All potentials are reported in volts.

For *n*-type semiconductors, like BiVO_4_, the conduction-band minimum lies at potentials more negative than V_fb_ [67]. Therefore, the E_CB_ values listed in Table 2, approximated as equal to V_fb_, represent upper (i.e., least negative) bounds for the conduction-band edge and range from +0.01 to +0.28 V vs. RHE. Because the measurements were performed on porous particulate films, the absolute values of V_fb_ and N_d_ should be regarded as semiquantitative estimates and are used mainly for comparison within the sample series. These values are in good agreement with previously reported values for monoclinic BiVO_4_ [68,69].

The donor densities obtained from the Mott–Schottky analysis ranged from 1.13 to 2.94 × 10^20^ cm^−3^. The relatively high values indicate substantial electronic carrier densities, which may contribute to improved electronic conductivity; however, their assignment specifically to oxygen vacancies should be considered tentative in the absence of independent defect characterization such as XPS, EPR, or complementary spectroscopic evidence [70] Likewise, a higher donor density should not be interpreted directly as evidence of lower recombination, because defect states can either facilitate carrier transport or act as recombination centers depending on their concentration and energetic position.

The observed trends indicate that hydrothermal temperature exerts a strong influence on the electronic properties, with a non-monotonic dependence. The intermediate temperature of 180 °C generally produced the most negative (cathodic) flat-band potentials and the highest donor densities within the corresponding pH series. In contrast, treatment at 200 °C generally resulted in lower donor densities. This behavior suggests that the hydrothermal temperature modifies the concentration and/or electronic contribution of donor-type defects. The decrease in donor density at 200 °C may be associated with changes in crystallization and defect relaxation; however, this interpretation should be regarded as a plausible explanation rather than a direct experimental demonstration. The pH produced secondary variations, indicating that the two synthesis variables jointly influence the electronic properties and that an intermediate-temperature synthesis window favors higher donor densities while preserving the monoclinic crystal structure.

The Nyquist plots for all samples are shown in Figure 5b. All photocatalysts exhibited a single depressed capacitive semicircle, indicating that the dominant electrochemical response can be described by a principal interfacial charge-transfer process. Because the diameter of the semicircle is related to the charge-transfer resistance (R_ct_), a smaller arc generally indicates lower interfacial resistance and more favorable charge-transfer kinetics [71]. Thus, comparison of the semicircle diameters provides complementary information on how the synthesis variables affect the semiconductor–electrolyte interface. The EIS spectra were fitted using the equivalent circuit: R_s_ + (R_ct_ ∥ CPE), where R_s_ represents the ohmic resistance associated primarily with the electrolyte and electrical contacts, R_ct_ represents the charge-transfer resistance at the semiconductor–electrolyte interface, and the constant phase element (CPE) accounts for non-ideal capacitive behavior associated with the electrical double layer and space-charge region [72,73]. The electrochemical parameters obtained from the fitting are listed in Table 3. The CPE exponents ranged from 0.95 to 0.98, indicating behavior close to ideal capacitive response with relatively small dispersion of relaxation times [73]. Although surface roughness and porosity may contribute to this non-ideal behavior, the present EIS data do not allow these contributions to be uniquely separated from other sources of interfacial heterogeneity.

The relatively low and homogeneous R_s_ values (9–22 Ω) indicate that the differences observed among the samples are unlikely to arise primarily from uncompensated ohmic resistance and are instead associated mainly with interfacial processes. The effective capacitances calculated using the Brug approximation were remarkably uniform across the entire set of samples (9.6–12.6 μF cm^−2^), suggesting that large differences in electrochemically accessible interfacial area are unlikely to be the dominant factor controlling the observed EIS response [74]. Therefore, the variations in R_ct_ are more reasonably interpreted in terms of differences in interfacial charge-transfer kinetics, although capacitance-derived surface-area comparisons should be treated cautiously for heterogeneous semiconductor electrodes.

The diameter of the semicircle, corresponding predominantly to R_ct_, is inversely related to the ease of charge transfer at the semiconductor–electrolyte interface. Thus, smaller semicircles indicate lower interfacial charge-transfer resistance and more favorable carrier-transfer kinetics [75]. The overall trends revealed a strong dependence on hydrothermal temperature, reproducing the non-monotonic behavior observed in the Mott–Schottky analysis. The materials synthesized at 180 °C formed a clearly differentiated low- R_ct_ group, with R_ct_ values 1.2–4.8 times lower than those obtained under the other synthesis conditions. In contrast, the samples synthesized at 160 °C exhibited the highest resistances in the series, with semicircles that did not fully close within the investigated frequency range.

Importantly, the lowest R_ct_ value was obtained for the material exhibiting the highest I(040)/I(110) ratio. This correlation suggests that the preferential crystallographic orientation and associated crystal habit may contribute to the interfacial charge-transfer behavior. However, the EIS data alone do not establish facet-mediated spatial separation of charge carriers. Rather, the result indicates that the crystallographic orientation/morphology identified by XRD and SEM is correlated with more favorable interfacial kinetics, possibly through differences in surface atomic configuration, adsorption, or charge-transfer pathways. This interpretation is also consistent with the relatively stable optical band gap and Raman fingerprint, indicating that the improved electrochemical response is unlikely to originate from the formation of a different BiVO_4_ polymorph.

It should be noted that EIS and chronoamperometry were recorded under an applied bias of 0.58 V vs. SCE (equivalent to +1.24 V vs. RHE at pH 7), selected to obtain measurable and reproducible signals from particulate films, whereas the photocatalytic tests were conducted under open-circuit conditions. The photoelectrochemical parameters are therefore used as comparative descriptors for ranking the samples rather than as direct measures of charge-transfer kinetics under photocatalytic conditions.

Transient chronoamperometry under intermittent illumination exhibited an immediate and reversible photocurrent response for all photocatalysts, with substantially lower dark currents than the photocurrents generated under illumination (Figure 6), confirming that the measured signal originates predominantly from photogenerated charge carriers. The steady-state photocurrent density reflects the combined contributions of charge generation, separation, transport, and extraction at the semiconductor–electrolyte interface. Therefore, its magnitude can be used as an indirect indicator of the efficiency with which photogenerated carriers reach the interface and participate in interfacial charge-transfer processes [76,77].

The overall trends closely reproduced the hierarchy established by electrochemical impedance spectroscopy, providing independent evidence that the samples with lower charge-transfer resistance also exhibited higher photocurrent densities. The photocatalysts synthesized at 180 °C again formed a clearly superior group, with steady-state photocurrent densities 1.6–4.3 times higher than those of the remaining samples (Table 4), further identifying the intermediate temperature as the most favorable condition within the investigated synthesis domain.

The superior photoelectrochemical response of the 180 °C materials can therefore be interpreted as the combined consequence of several experimentally supported characteristics: (i) their favorable preferential crystallographic orientation, reflected by the high I(040)/I(110) ratios; (ii) their plate-like crystal habit observed by SEM, particularly for M5 and M6; (iii) their relatively high donor densities obtained from Mott–Schottky analysis; (iv) their lower interfacial charge-transfer resistance; and (v) their higher photocurrent densities under illumination. Rather than attributing the response exclusively to a particular exposed facet, these results support a broader structure–electronic–property relationship in which crystallographic orientation, morphology, and carrier transport collectively influence the photoelectrochemical behavior [78,79].

In contrast, the materials synthesized at higher temperatures exhibited quasi-rectangular transient responses with minimal current decay, suggesting relatively stable photocurrent generation during each illumination period but a lower overall steady-state carrier flux. The lower-temperature materials, in turn, combined lower photocurrent densities with a progressive increase in photocurrent from cycle to cycle. This behavior may indicate gradual changes in surface charge accumulation or interfacial equilibration during repeated illumination cycles; however, further measurements would be required to assign this response to a specific surface process.

Taken together, the optical and electrochemical characterization establishes that the principal effect of the synthesis variables is not a substantial modification of the BiVO_4_ band gap or crystal phase, but rather a modulation of carrier density and interfacial charge-transfer kinetics. The optimum response at 180 °C coincides with the morphological and crystallographic features identified by SEM and XRD, respectively, providing a consistent link between synthesis conditions, crystal development, electronic properties, and photoelectrochemical performance.

### 2.5. Photocatalytic Activity

Before irradiation, the suspensions were kept in the dark for 45 min to establish adsorption–desorption equilibrium between methylene blue and photocatalyst surfaces (Figure 7). During this stage, the apparent decrease in dye concentration ranged from 11.5% to 17.1%, indicating that adsorption contributed appreciably to the initial dye removal. The substantially greater dye removal observed under irradiation compared with the dark conditions confirms the predominant contribution of photocatalysis to the overall MB removal process. In the absence of catalyst, MB photolysis under the same irradiation conditions produced only 5.2% discoloration after 90 min (Figure 7a), indicating that dye self-sensitization makes a negligible contribution. It should be emphasized that the decrease in absorbance at 665 nm reflects discoloration of the chromophore and does not demonstrate mineralization [80,81]. During the 90 min of irradiation, all samples exhibited a progressive decrease in MB concentration, but at markedly different rates (Figure 7 and Table 5). Taking *C*_0_ as the concentration measured at the end of the dark adsorption–desorption period, the M5 sample achieved 86.8% discoloration, while the M6 and M8 samples reached 80.2% and 76.3%, respectively. In contrast, the M1 sample achieved only 28.7% discoloration. At all pH levels, photocatalytic activity increased from 160 to 180 °C and decreased at 200 °C, indicating that 180 °C provides the most favorable synthesis condition within the investigated experimental domain and is consistent with the maximum preferential crystallographic orientation observed under this condition.

The effects of the two synthesis factors on *k_app_* were first evaluated by two-way ANOVA (Table S1). Because the 3^2^ design contains one observation per condition, the pH × T interaction was used as the error term and tested separately with Tukey’s one-degree-of-freedom test for non-additivity. Both factors significantly affected *k_app_* (pH: 58.1% of the total sum of squares, *p* = 0.002; temperature: 39.3%, *p* = 0.004), without significant non-additivity (*p* = 0.12). A quadratic response-surface model in coded variables (Table S3) described *k_app_* well (R^2^ = 0.975; adjusted R^2^ = 0.932), with significant quadratic terms for pH (*p* = 0.011) and temperature (*p* = 0.010) and a non-significant pH × T interaction term (*p* = 0.80). Its stationary point (pH = 0.58, T =183 °C) lies inside the experimental domain, next to sample M5, confirming that the maximum activity is reached at intermediate pH and temperature.

A linear correlation analysis based on Pearson’s correlation coefficient (*r*) was performed between *k_app_* and each structural, optical, and electrochemical descriptor to identify which properties varied systematically with photocatalytic activity and which were largely independent of it (Table S2). Because the nine samples shared the same crystalline phase and exhibited nearly invariant V–O bond lengths, unit-cell parameters, and crystallite sizes, these parameters are unlikely to be the primary factors governing the differences in photocatalytic activity. Similarly, the optical band-gap energy showed no statistically significant correlation with *k_app_* (*r* = −0.384, *p* = 0.31). The structural descriptor most strongly associated with photocatalytic activity was the I(040)/I(110) ratio (*r* = 0.931, *p* < 0.001), indicating that samples with greater preferential crystallographic orientation along the [010] direction also exhibited higher photocatalytic activity. This correlation remained significant after Bonferroni correction for nine descriptors (*p* = 0.002) and in a permutation test (*p* < 0.001), was confirmed by Spearman rank correlation (ρ = 0.867, *p* = 0.002), and did not depend on any single sample: removing M5, the most active sample, still yielded *r* = 0.888 (*p* = 0.003). Together with the SEM observations of plate-like particles in samples M5 and M6, this suggests that crystal habit and preferential orientation contribute to the photocatalytic response.

The three electrochemical characterization techniques converged on the same overall hierarchy. *k_app_* was negatively correlated with R_ct_ (*r* = −0.763, *p* = 0.017) and positively correlated with the steady-state photocurrent density (*r* = 0.773, *p* = 0.015). Because these correlations do not remain significant after Bonferroni correction, they are regarded as consistent trends rather than independent evidence. The ANOVA also showed that pH accounts for most of the variance in *k_app_*, whereas temperature dominates the variance in I(040)/I(110) (Table S1). Preferential orientation is therefore an important, but not exclusive, descriptor of activity; pH-dependent factors not captured by I(040)/I(110), such as surface termination or adsorption, likely contribute as well. The joint trends in I(040)/I(110), R_ct_, photocurrent, and *k_app_* are consistent with, but do not prove, the {010}/{110} charge-separation mechanism demonstrated for faceted BiVO_4_ crystals [82,83].

The specific surface area was not measured by N_2_ physisorption. Nevertheless, several observations indicate that surface area is not the dominant factor. The effective capacitance obtained by EIS, a proxy for the electrochemically accessible area, varied by only ±13% (9.6–12.6 μF cm^−2^), and dark MB adsorption, a measure of the dye-accessible surface, varied be only 1.5-fold (11.5–17.1%), whereas *k_app_* varied six-fold. The correlation between I(040)/I(110) and *k_app_* remained high after controlling for dark adsorption (partial *r* = 0.860, *p* = 0.006) or for the effective capacitance (partial *r* = 0.938, *p* = 0.001; Table S2). In addition, the SEM images (Figure 2) do not show smaller primary particles for the most active samples than for the least active ones, which further argues against a dominant surface-area effect. Nevertheless, BET measurements would provide a more direct normalization.

The reusability of the most active sample, M5, was evaluated over four consecutive cycles under identical conditions, with a washing step between cycles (Figure 8). The discoloration efficiency decreased only slightly, from 86.9% in the first cycle to 85.7%, 84.9%, and 84.1% in the second, third, and fourth cycles, respectively, corresponding to a retention of 96.8% of the initial activity. The first-cycle value agrees with that of the independent experiment reported in Table 5 (86.8%), supporting the reproducibility of the photocatalytic test. Dark adsorption also decreased slightly, from 17.1% to 15.1%, suggesting that the small loss of activity is mainly associated with minor catalyst losses during recovery rather than substantial catalyst deactivation. Such cycling tests are a common criterion for assessing the practical stability of photocatalysts [84,85]. Structural characterization of the used catalyst was not performed.

To identify the reactive species involved in methylene blue decolorization, selective scavengers were evaluated using the most active sample (0.50–180 °C; Figure 9a). Benzoquinone (BQ) was used as a scavenger for superoxide radicals $\left({•O}_{2}^{-}\right)$, EDTA for photogenerated holes (*h*^+^), and isopropanol (IPA) for hydroxyl radicals (•OH) [86]. BQ produced the strongest inhibition, decreasing MB decolorization from 86.8% to 26.8%, which indicates that electron consumption by dissolved O_2_ is essential for the process. EDTA decreased decolorization to 61.4%, indicating a significant contribution from photogenerated holes, whereas IPA produced the weakest effect (78.5%, approximately 10% suppression), suggesting that •OH plays a comparatively minor role. The inhibition sequence was therefore BQ > EDTA > IPA.

These results can be compared with the band-edge positions estimated for sample M5 (Table 2). Taking V_fb_ (+0.01 V vs. RHE) as an upper bound of E_CB_, the driving force for the one-electron reduction of O_2_ to ${•O}_{2}^{-}$ (+0.08 V vs. RHE at pH 7) would be at least 70 mV and probably larger, since the conduction-band minimum of an *n*-type semiconductor lies at potentials more negative than V_fb_ [67,87,88]. Because this margin is within the uncertainty of Mott–Schottky analysis on nanostructured particulate films, and because scavenger results require cautious interpretation, the BQ effect is not assigned exclusively to ${•O}_{2}^{-}$ [67,89]. The multi-electron reduction of O_2_ to H_2_O_2_ (+0.68 V vs. RHE) is thermodynamically more favorable and may also contribute [88]. Direct reduction of MB to its colorless leuco form (+0.42 V vs. RHE) is also thermodynamically possible and is partially reversed by dissolved O_2_ [90]; moreover, dye-sensitized reductive bleaching under visible light can cause discoloration without true photocatalytic degradation [91]. Both effects are further reasons to report decolorization rather than degradation. The valence-band position (+2.43 V vs. RHE) provides holes with sufficient oxidizing power for direct MB oxidation but lies below the potential of the •OH/H_2_O couple (+2.73 V vs. RHE), so that direct oxidation of water to •OH is thermodynamically unfavorable (Figure 9b). This is consistent with the minor effect of IPA; the small •OH contribution may instead arise from the reductive decomposition of photogenerated H_2_O_2_ (H_2_O_2_ + *e*^−^→ •OH + OH^−^). EPR spin trapping would be required to identify the radical species unambiguously [92].

The higher activity of sample M5 may be associated with the crystallographic and morphological characteristics of the material. In monoclinic BiVO_4_, photogenerated electrons have been shown to accumulate preferentially on {010} facets, and the preferential orientation associated with the {010} family is maximized in sample M5, as indicated by its highest I(040)/I(110) ratio [46,48,82,83,93]. Accordingly, the enhanced photocatalytic activity of sample M5 may result from more favorable carrier availability at the reactive surface, facilitating electron transfer to dissolved oxygen. However, this interpretation remains a mechanistic hypothesis supported by the combined XRD, SEM, EIS, photocurrent, and scavenger results; facet-specific electron accumulation was not verified directly for the present samples.

Based on the results presented above, the photocatalytic process can be tentatively described by the following sequence of reactions. Upon absorption of photons with energy equal to or greater than the optical band-gap energy, electron–hole pairs are generated (Equation (4)). As a working hypothesis, these photogenerated carriers are proposed to undergo spatially differentiated transfer toward surfaces associated with the {010} and {110} orientations, respectively (Equation (5)). Electrons associated with {010}-related surfaces can reduce dissolved oxygen to superoxide radicals (Equation (6)), consistent with the strong inhibition observed upon addition of benzoquinone. Meanwhile, photogenerated holes associated with {110}-related surfaces can directly oxidize adsorbed methylene blue molecules (Equation (7)), consistent with the inhibition observed upon the addition of EDTA. A minor fraction of hydroxyl radicals may be generated by the reduction of photogenerated H_2_O_2_ by conduction-band electrons (Equation (8)), consistent with the relatively weak effect of isopropanol (Figure 9a); direct oxidation of water by valence-band holes is thermodynamically unfavorable because the valence band of sample M5 lies below the •OH/H_2_O couple potential (Figure 9b).

(4) $${\mathrm{BiVO}}_{4}+\mathrm{hv}\to e_{\mathrm{CB}}^{-}+h_{\mathrm{VB}}^{+}$$

(5) $$e_{\mathrm{CB}}^{-}\to \{010\};h_{\mathrm{VB}}^{+}\to \{110\}$$

(6) $$e^{-}\left\{010\right\}+O_{2}\to {•O}_{2}^{-};{•O}_{2}^{-}+\mathrm{MB}\to \mathrm{oxidation products}$$

*h*^+^{110} + *MB*(*ads*) → by products (7)

(8) $$e_{\left(\mathrm{CB}\right)}^{-}+H_{2}O_{2}\to •\mathrm{OH}+{\mathrm{OH}}^{-};•\mathrm{OH}+\mathrm{MB}\to \mathrm{oxidation products}$$

## 3. Materials and Methods

### 3.1. Materials and Reagents

The reagents used in this study were of analytical grade and were used as received without further purification unless otherwise stated. Bismuth nitrate pentahydrate, Bi(NO_3_)_3_·5H_2_O (Sigma-Aldrich, St. Louis, MO, USA, 98% purity), and ammonium metavanadate, NH_4_VO_3_ (Merck, Darmstadt, Germany, 99% purity), were used as the bismuth and vanadium precursors, respectively. Nitric acid (HNO_3_, Merck, 65% purity) and ammonia solution (NH_3_, Merck, 25% purity) were used to adjust the acidity of the reaction medium and control the pH, respectively. Milli-Q water (Merck Millipore, Burlington, MA, USA) was used throughout the synthesis and washing procedures.

### 3.2. Hydrothermal Synthesis of BiVO_4_

In a typical synthesis (Scheme 1), 3 mmol of Bi(NO_3_)_3_·5H_2_O and 3 mmol of NH_4_VO_3_ were added under sonication to 30 mL of 4 M aqueous HNO_3_ solution. After complete dissolution of the precursors, the pH of the resulting solution was adjusted to the desired value by dropwise addition of the NH_3_ solution under continuous stirring. The resulting suspension was vigorously stirred for 2 h at room temperature and subsequently transferred to a 50 mL stainless-steel autoclave fitted with a Teflon liner (Huanyu, China). The suspension was then subjected to hydrothermal treatment at the selected temperatures for 12 h.

After hydrothermal treatment, the resulting solid was recovered by filtration using 0.45 μm cellulose membrane filters and repeatedly washed with Milli-Q water until the pH of the filtrate reached approximately 7. The recovered material was finally dried at 90 °C for 12 h.

The effects of reaction pH and hydrothermal treatment temperature on BiVO_4_ synthesis were systematically evaluated using a complete 3^2^ factorial design [37]. The two experimental factors, namely reaction pH and hydrothermal temperature, were varied at three levels each, resulting in a matrix of nine synthesis conditions. All BiVO_4_ samples were prepared without the use of structure-directing agents. The experimental conditions and corresponding sample labels are summarized in Table 6.

### 3.3. Semiconductor Characterization

The structural properties of the synthesized materials were investigated using X-ray diffraction (XRD) and Raman spectroscopy. XRD patterns were recorded using a D8 Advance diffractometer (Bruker, Karlsruhe, Germany) equipped with Cu K_α1_ radiation (λ = 1.540598 Å) over a 2θ range of 10–90°. Raman spectra were acquired using a Cora 5001 spectrometer (Anton Paar, Graz, Austria) equipped with a 780 nm excitation laser. Fourier-transform infrared (FTIR) spectra were recorded using an FT/IR-6X spectrometer (Jasco, Tokyo, Japan) equipped with an attenuated total reflectance (ATR) accessory, over the wavenumber range of 4000–400 cm^−1^.

Diffuse reflectance spectra were obtained using a UV–Vis Evolution 220 spectrophotometer (Thermo Scientific, Waltham, MA, USA) equipped with an integrating sphere, over the wavelength range of 300–800 nm.

For electrochemical characterization, photoanodes with an active area of 1 cm^2^ were fabricated by depositing the BiVO_4_ samples onto indium tin oxide (ITO)-coated glass substrates using the doctor-blade technique. A saturated calomel electrode (SCE) was used as the reference electrode, while a graphite rod served as the counter electrode. A 0.5 M Na_2_SO_4_ aqueous solution with a pH close to 7 was used as the supporting electrolyte. Electrochemical measurements were performed using an IFC1000 potentiostat (Gamry Instruments, Warminster Township, PA, USA).

The electronic properties of the photoanodes were evaluated by Mott–Schottky analysis. The measurements were performed in the dark at a single frequency of 500 Hz with an AC amplitude of 10 mV rms, scanning the DC potential from –0.60 to +0.60 V vs. SCE in 25 mV steps. The corresponding plots were obtained by representing the inverse square of the space-charge capacitance $\left(1/C_{\mathrm{SC}}^{2}\right)$ as a function of the applied potential.

Electrochemical impedance spectroscopy (EIS) measurements were performed under visible-light irradiation at a constant potential of 0.58 V versus SCE, using a sinusoidal perturbation with an amplitude of 10 mV RMS. The impedance spectra were recorded over a frequency range of 20 kHz to 0.1 Hz.

The photoelectrochemical response of the photoanodes was evaluated by chronoamperometry at a constant potential of 0.58 V versus SCE under alternating visible-light irradiation and dark conditions. The photocurrent density was monitored as a function of time during successive light-on/light-off cycles.

### 3.4. Photocatalytic Activity Experiments

Photocatalytic discoloration experiments were conducted at room temperature (approximately 25 °C) in a batch-type quartz reactor. A 20 W white-light LED lamp (Mercury, Bogotá, Colombia; correlated color temperature 6500 K; luminous flux 1800 lm) was used as the irradiation source and positioned 10 cm above the reactor. For each experiment, 20 mg of BiVO_4_ photocatalyst was dispersed in 50 mL of an aqueous methylene blue (MB) solution with an initial concentration of approximately 10 mg L^−1^.

Before irradiation, the suspension was continuously stirred in the dark for 45 min to establish adsorption–desorption equilibrium. The photocatalytic reaction was initiated by switching on the LED lamp. Aliquots were withdrawn at 10 min intervals during irradiation and centrifuged to remove suspended photocatalyst particles before analysis. The residual MB concentration was determined using a UV-Vis Evolution 200 spectrophotometer (Thermo Scientific) by monitoring the absorbance at λ_max_ = 665 nm. A photolysis control (MB solution irradiated without a catalyst) and a dark adsorption control were carried out under identical conditions. Each experiment was performed in triplicate using independently prepared suspensions; the error bars in Figure 7 correspond to the standard deviations, and the uncertainty of *k_app_* in Table 5 corresponds to the standard deviation among replicates. For the scavenger tests, benzoquinone (2 mM), EDTA (5 mM), or isopropanol (2.5 mL L^−1^) was added to the suspension before the dark period.

The photocatalytic discoloration kinetics were evaluated using the pseudo-first-order approximation derived from the Langmuir–Hinshelwood model according to Equation (9):

(9) $$\ln\left(\frac{C_{t}}{C_{0}}\right)\approx -k_{\mathrm{app}}t$$

where *C*_0_ is the initial MB concentration, *C_t_* is the MB concentration at irradiation time t, and *k_app_* is the apparent pseudo-first-order rate constant. The apparent rate constant represents the overall observed photocatalytic reaction rate and is influenced by several processes, including pollutant adsorption, photogeneration and transport of charge carriers, interfacial charge transfer, and subsequent surface redox reactions [94].

Reusability was evaluated for sample M5 over four consecutive cycles. After each cycle, the catalyst was recovered by centrifugation, washed with deionized water, and resuspended directly in fresh MB solution for the next cycle.

### 3.5. Statistical Analysis

The effects of pH and temperature were evaluated by a two-way ANOVA using the pH × T interaction as the error term (4 degrees of freedom), complemented by Tukey’s one-degree-of-freedom test for non-additivity. A full quadratic response-surface model was fitted in coded variables (*x*_1_ = (pH − 0.50)/0.25; *x*_2_ = (T − 180)/20). Pearson and Spearman correlation coefficients between *k_app_* and each descriptor were calculated, and *p*-values were corrected for multiple comparisons by the Bonferroni method (*m* = 9). The robustness of the correlations was assessed by leave-one-out analysis, a permutation test, bootstrap confidence intervals, and partial correlations controlling for dark adsorption and effective capacitance. All calculations were performed in R (version 4.3.3).

### 3.6. Use of Illustration Software and Generative AI Tools

Scheme 1 was drawn by the authors in BioRender (Science Suite Inc., Toronto, ON, Canada) using its library of predefined scientific icons; no generative AI function was used to create it. ChatGPT (OpenAI, GPT-5.6 Thinking) was used to improve the English language of the manuscript.

## 4. Conclusions

Using a full factorial 3^2^ design, the interactive influence of initial pH and hydrothermal temperature on the synthesis of BiVO_4_ without structure-controlling agents was systematically evaluated. All synthesis conditions yielded phase-pure monoclinic scheelite BiVO_4_, with unit-cell parameters, V–O bond length, crystallite size, and optical band-gap energy remaining virtually invariant across the investigated synthesis domain. This behavior indicates that the bulk structural and optical properties remained stable within the studied reaction window. In contrast, the degree of preferential crystallographic orientation with the {010} family, described by the I(040)/I(110) ratio, exhibited a pronounced non-monotonic dependence on the synthesis variables, reaching its maximum under hydrothermal treatment at pH 0.50 and 180 °C. This structural trend was consistent with the SEM observations, in which the samples synthesized at 180 °C and intermediate-to-high pH exhibited the most developed plate-like crystal habits.

Photoelectrochemical characterizations, including Mott–Schottky analysis, electrochemical impedance spectroscopy (EIS), and chronoamperometry, together with photocatalytic tests under visible-light irradiation, showed consistent trends across the investigated materials. Preferential orientation described by the I(040)/I(110) ratio was strongly correlated with the apparent rate constant (*r* = 0.931), and higher values were associated with lower interfacial charge-transfer resistance *R_ct_* and higher photocurrent density. The M5 sample (pH 0.50, 180 °C) achieved 86.8% MB discoloration after 90 min, with an apparent rate constant of *k_app_* = 0.0224 *min*^−1^, approximately six times higher than that of the least active material. Reactive-species scavenging experiments indicated that the discoloration proceeds mainly through O_2_ reduction pathways and direct oxidation by photogenerated holes (h^+^), with a comparatively minor contribution from hydroxyl radicals (•OH).

The combined XRD, SEM, PL, Mott–Schottky, EIS, photocurrent, and scavenger results are consistent with a facet-dependent charge-transfer mechanism involving surfaces associated with the {010} and {110} orientations. Within this interpretation, photogenerated electrons are proposed to preferentially participate in oxygen reduction at {010}-related surfaces, while photogenerated holes contribute to pollutant oxidation at {110}-related surfaces. However, the present measurements provide indirect evidence for this spatial charge-transfer model rather than direct visualization of facet-specific carrier separation. Future work will include facet-resolved measurements (selective photodeposition or surface photovoltage), EPR spin-trapping studies, open-circuit EIS and IPCE measurements, BET surface area determination, TOC analysis, and post-use catalyst characterization.

The photocatalytic efficiency of monoclinic scheelite BiVO_4_ was strongly associated with preferential crystallographic orientation and interfacial charge-transfer kinetics, whereas variations in optical band-gap energy and the principal bulk structural parameters were comparatively small across the synthesis domain. The strong correlation between I(040)/I(110) and *k_app_*, together with the corresponding trends in *R_ct_* and photocurrent density, identifies preferential crystallographic orientation as an important structural descriptor of photocatalytic performance under the investigated conditions.

Finally, this work defines a surfactant-free synthesis window centered at pH 0.50 and 180 °C that produces monoclinic BiVO_4_ with a highly developed preferential orientation and favorable photoelectrochemical and photocatalytic properties, providing a basis for the controlled synthesis of BiVO_4_ materials for photocatalytic and photoelectrochemical applications.

## Figures and Tables

**Figure 1:**
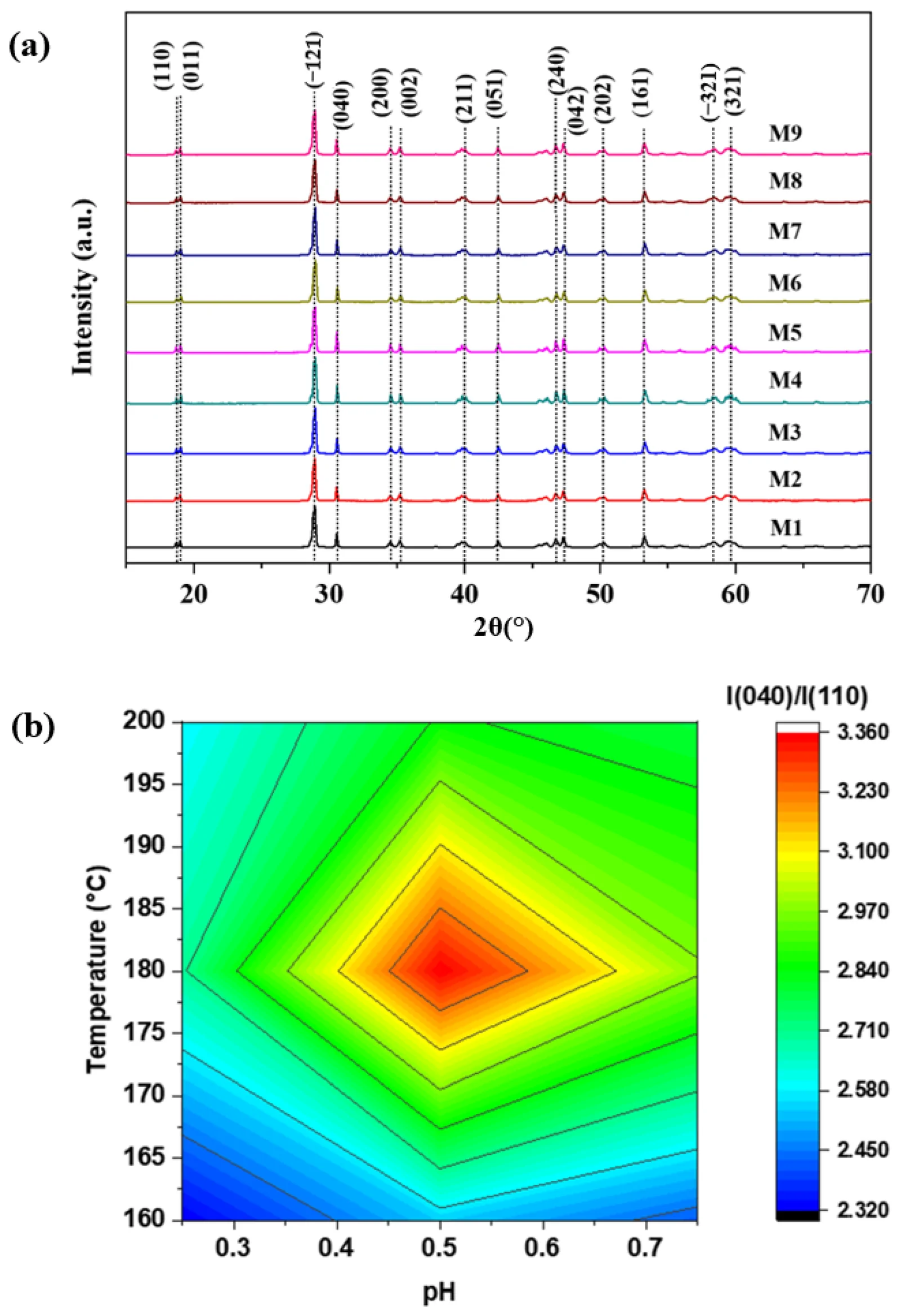
(**a**) XRD patterns of the synthesized BiVO_4_ samples. (**b**) I(040)/I(110) preferential crystallographic orientation descriptor as a function of initial pH and hydrothermal treatment temperature.

**Figure 2:**
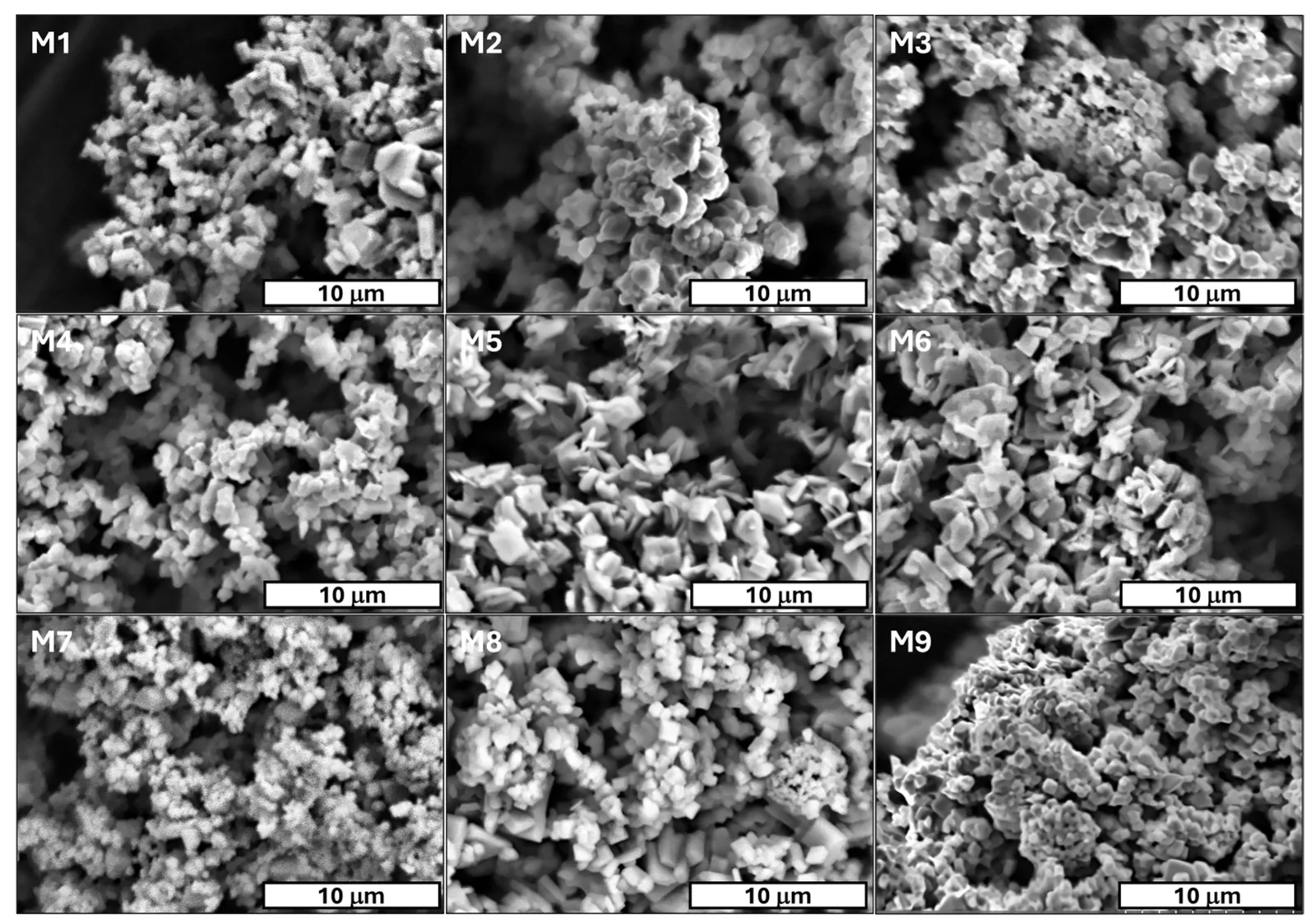
SEM micrographs of the nine BiVO_4_ samples synthesized by the hydrothermal method, arranged according to initial pH (columns) and hydrothermal treatment temperature (rows).

**Figure 3:**
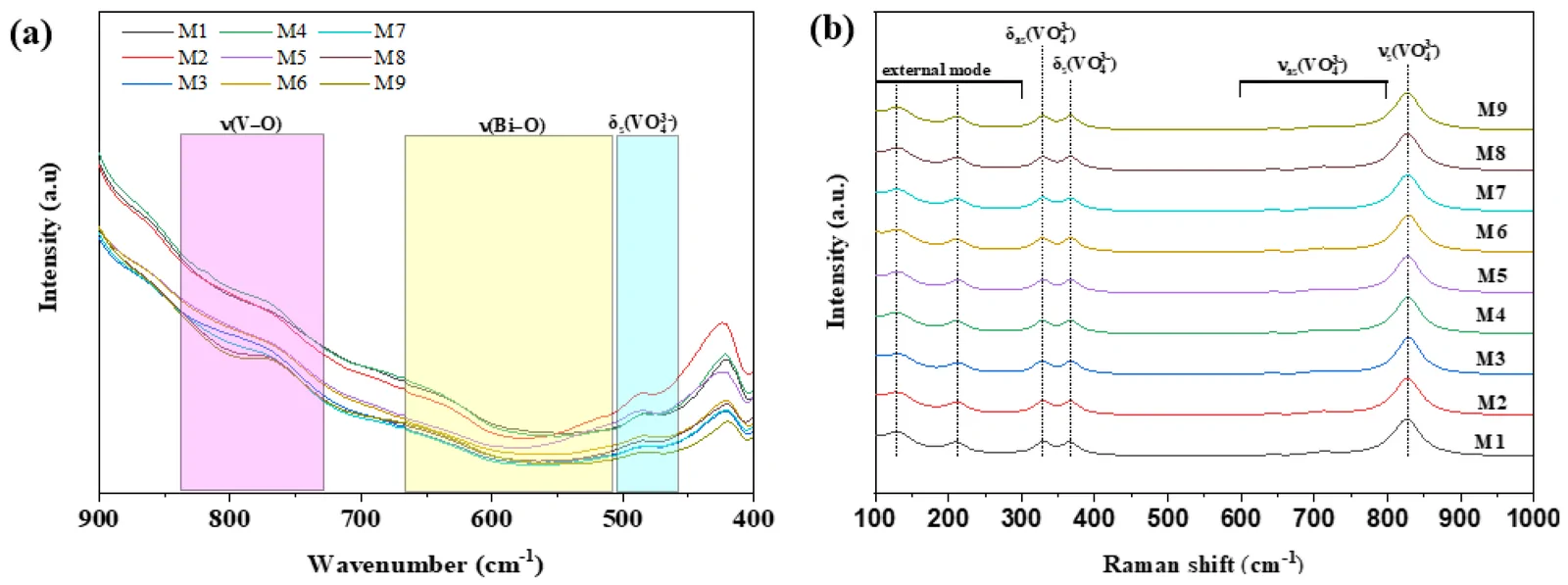
(**a**) FTIR spectra and (**b**) Raman spectra of the synthesized BiVO_4_ samples.

**Figure 4:**
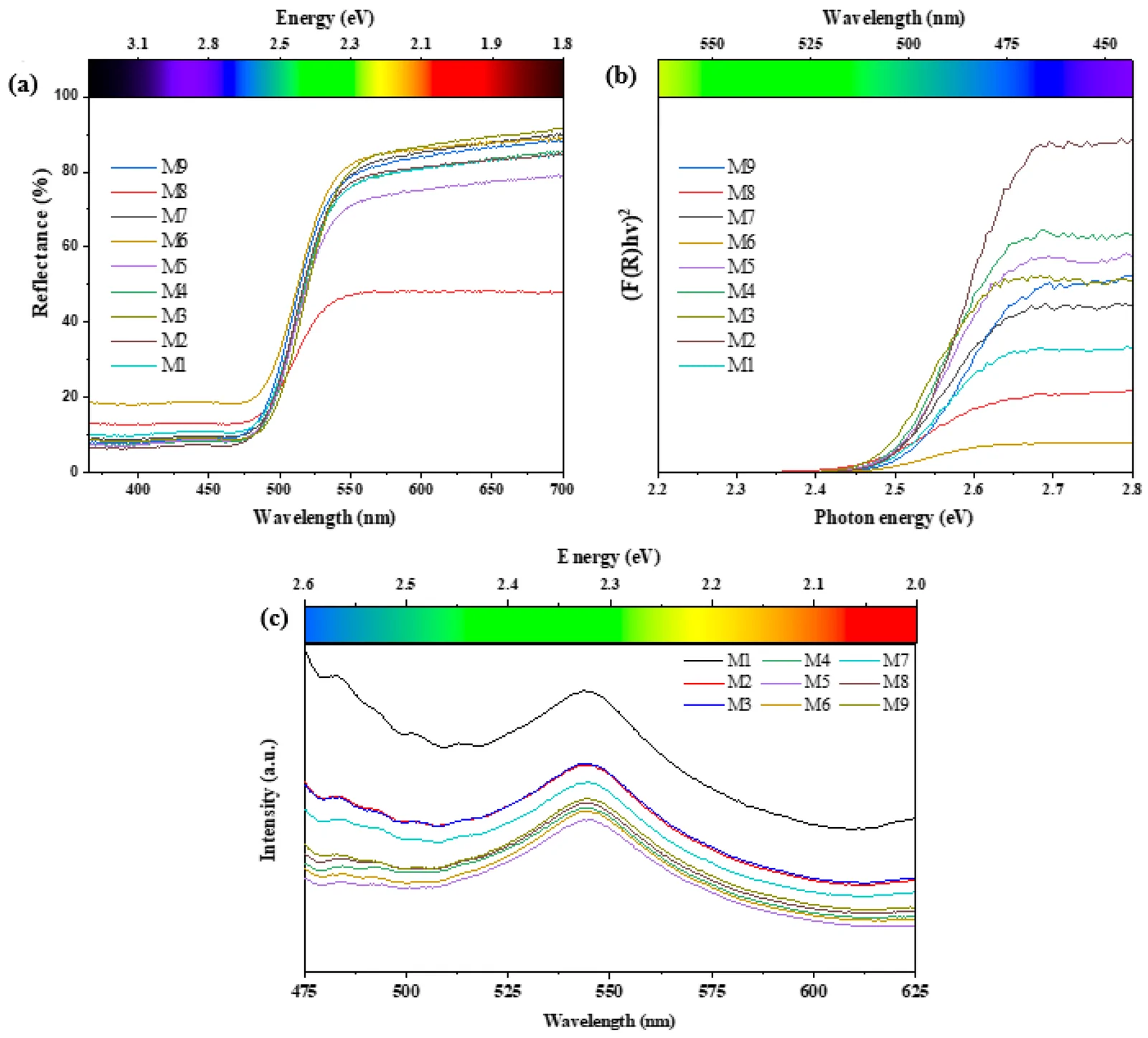
(**a**) UV-Vis diffuse reflectance spectra, (**b**) Tauc plots for band-gap estimation, and (**c**) photoluminescence (PL) spectra of the synthesized BiVO_4_ samples.

**Figure 5:**
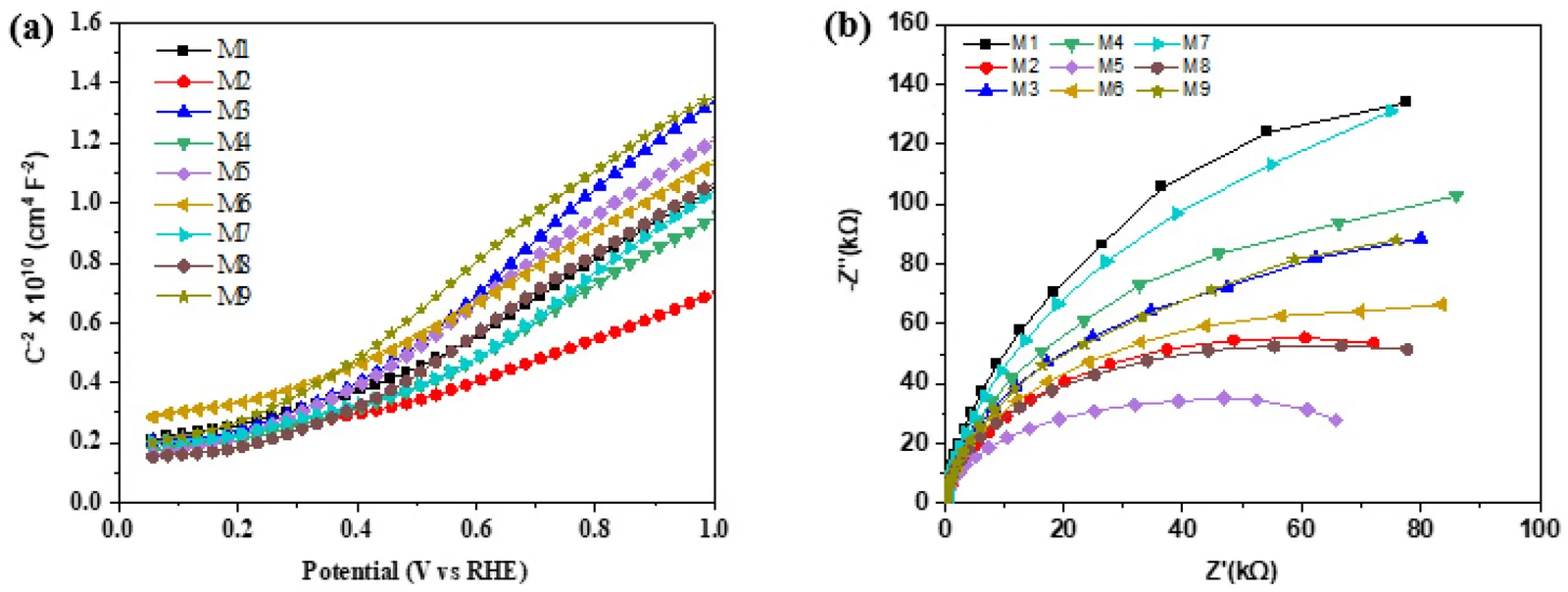
(**a**) Mott–Schottky plots and (**b**) Nyquist plots of the synthesized BiVO_4_ samples obtained by electrochemical impedance spectroscopy (EIS).

**Figure 6:**
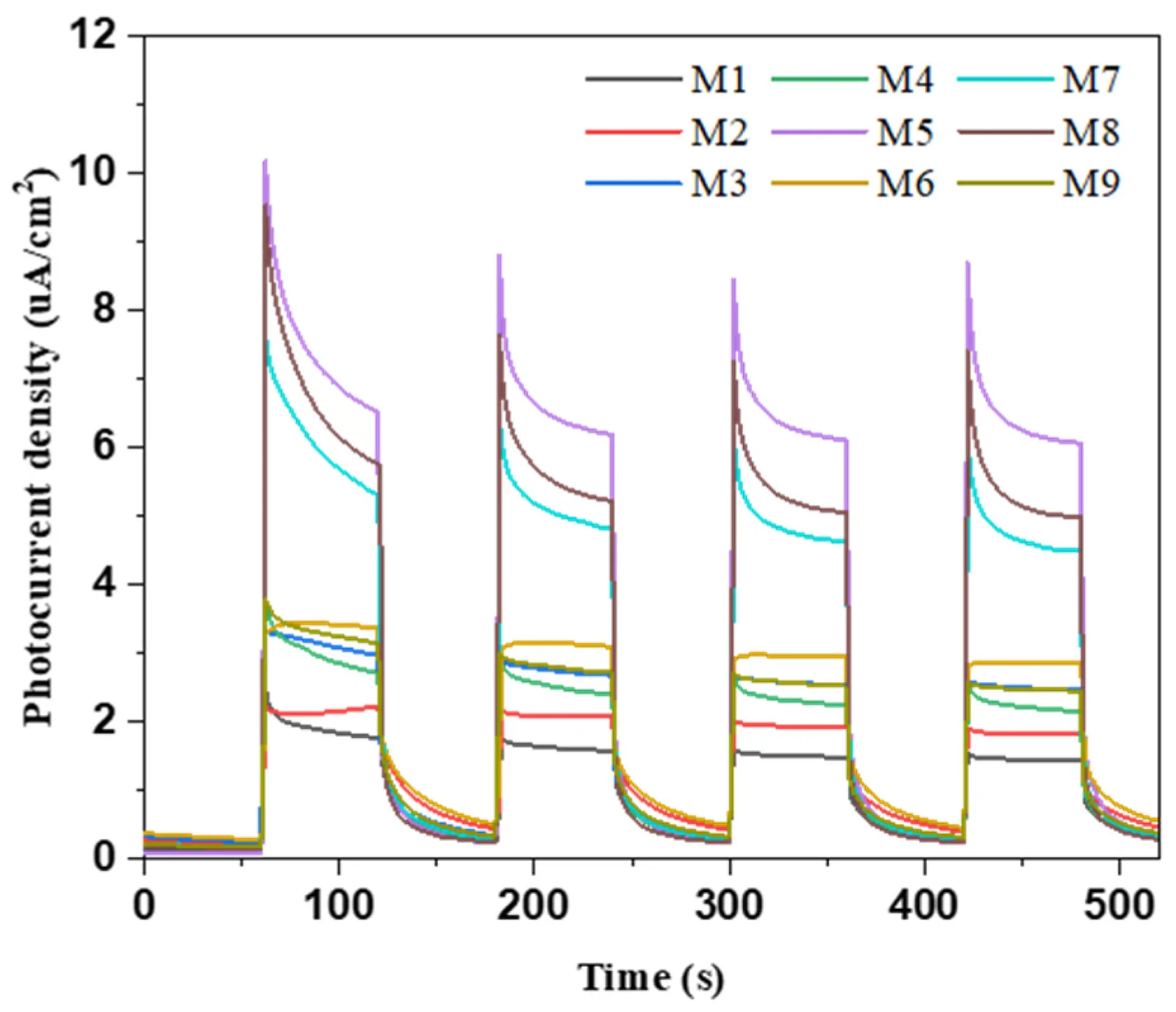
Chronoamperometry response of the BiVO_4_ synthesized samples under alternating light and dark conditions.

**Figure 7:**
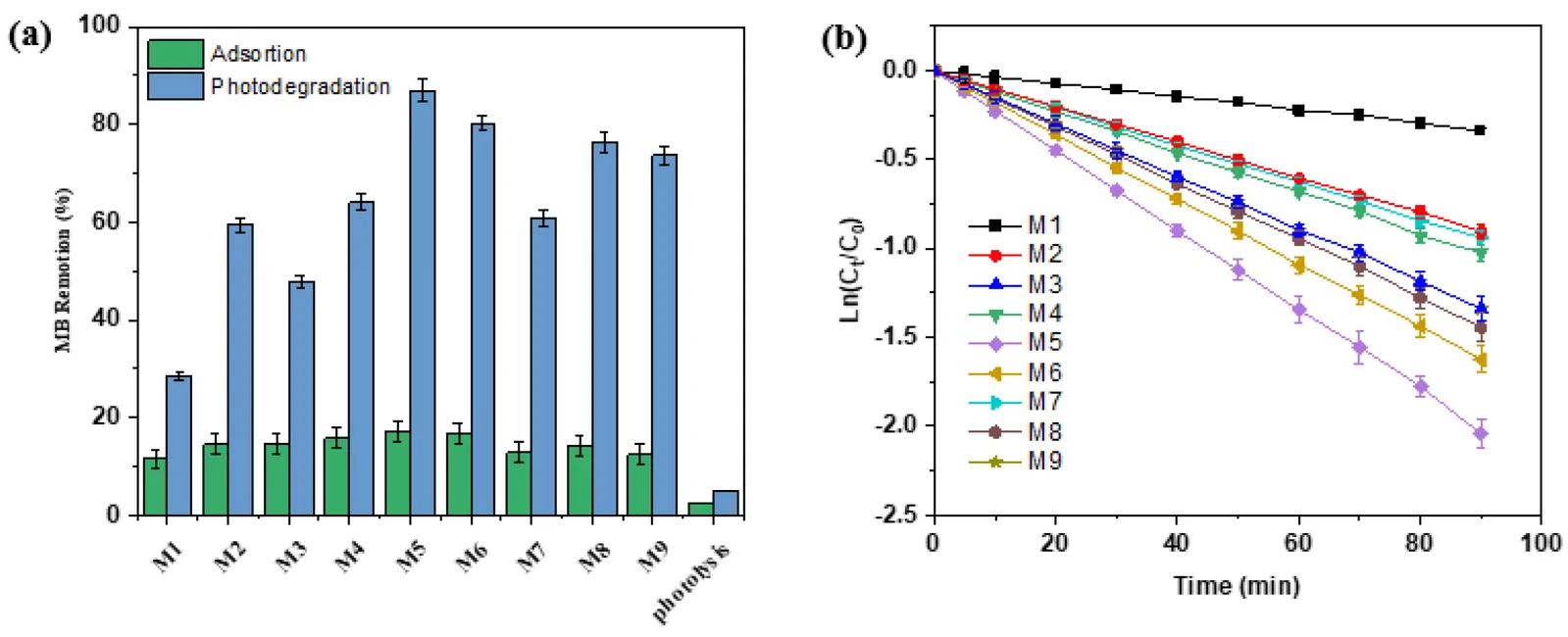
(**a**) MB adsorption in the dark followed by photocatalytic discoloration under visible-light irradiation and (**b**) pseudo-first-order kinetic fitting of MB discoloration for the nine synthesized BiVO_4_ samples.

**Figure 8:**
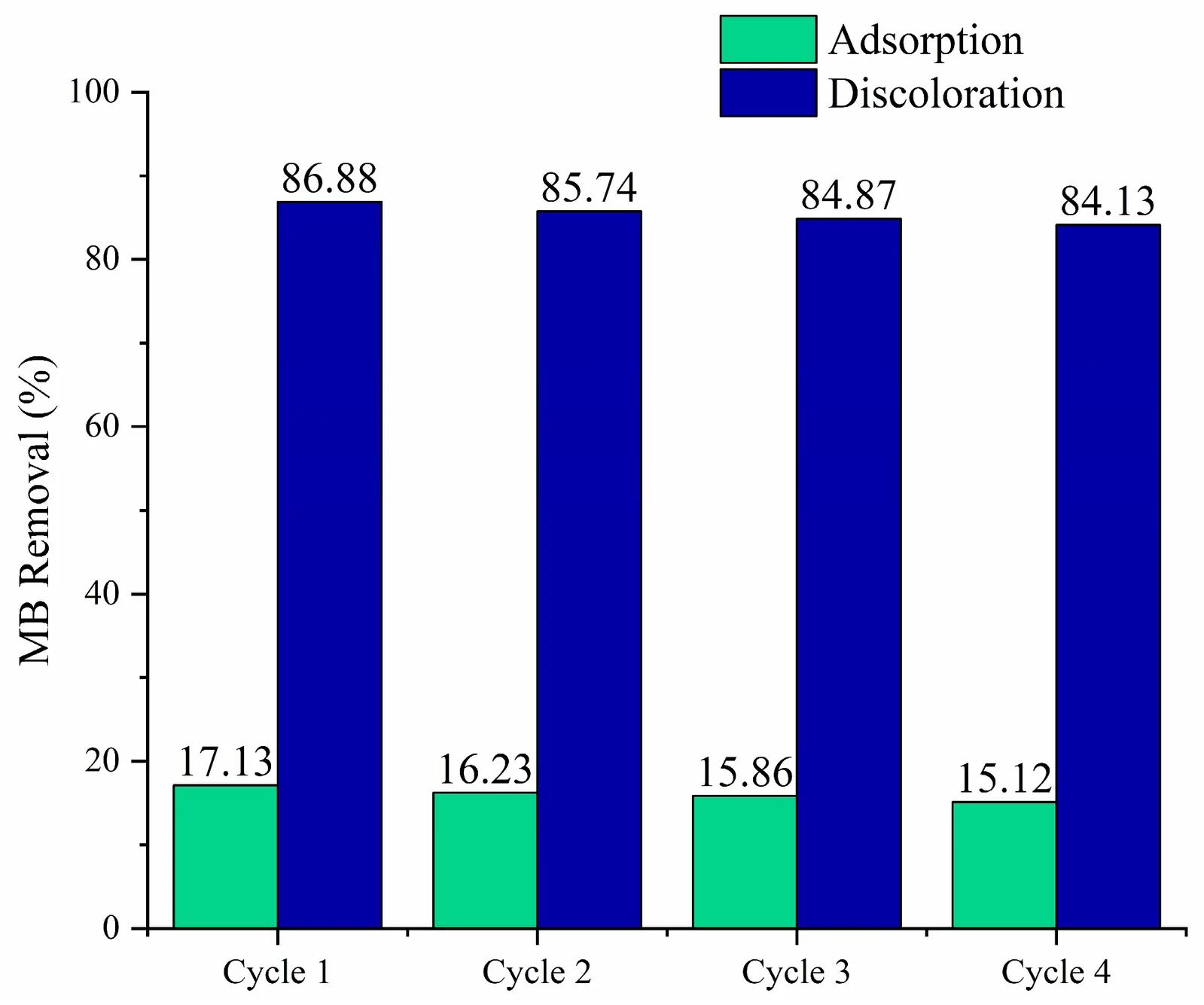
Reusability of M5 over four consecutive cycles: dark adsorption (45 min) and photocatalytic discoloration of MB after 90 min of visible-light irradiation.

**Figure 9:**
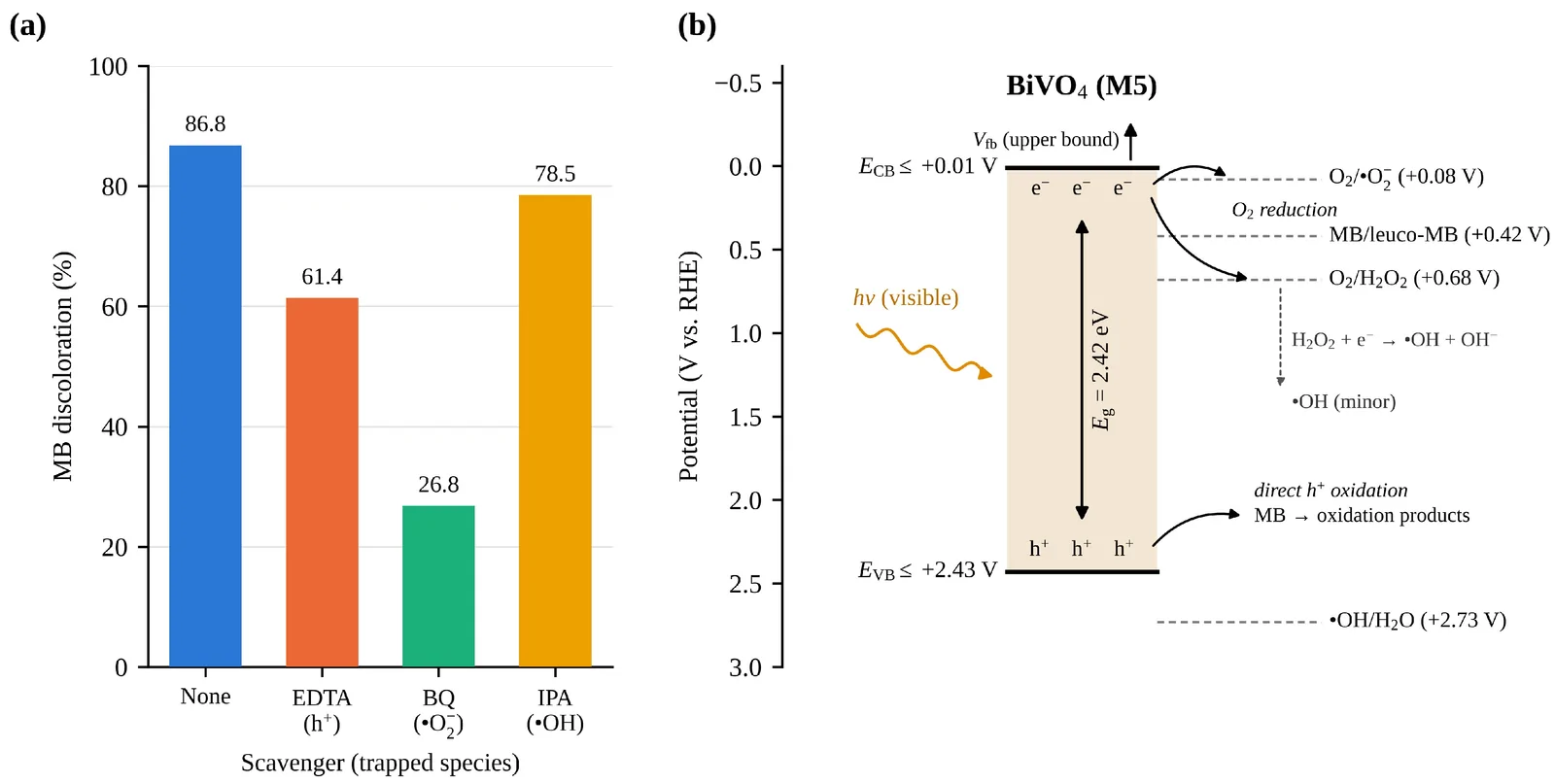
(**a**) Scavenger experiments for identifying the reactive species involved in MB photocatalytic discoloration and (**b**) proposed energy-level diagram for the photocatalytic discoloration of MB over BiVO_4_ samples.

**Scheme 1:**
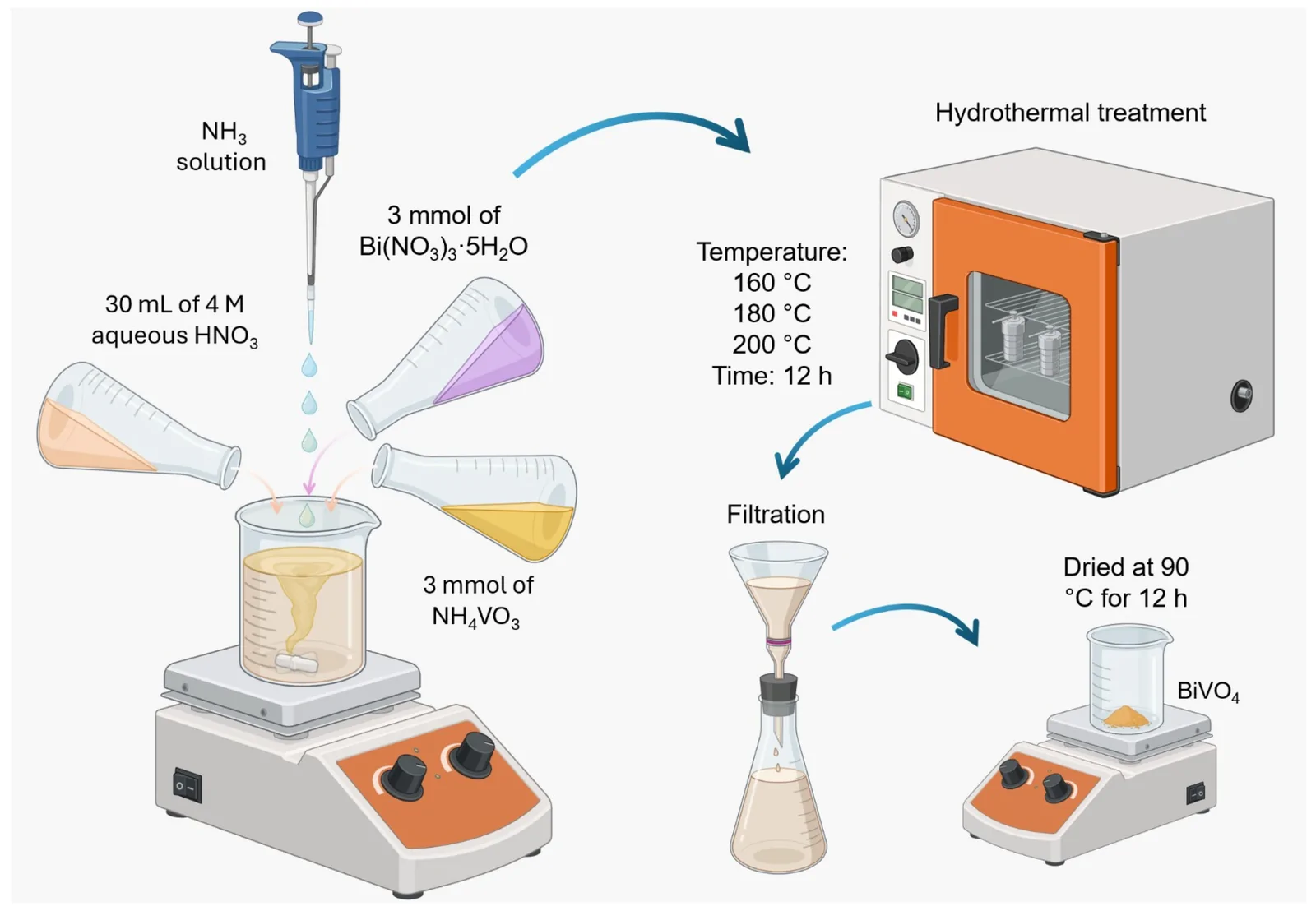
Experimental synthesis of the samples to obtain BiVO_4_. Created in BioRender. Millán-Franco, M.A. (2026). https://BioRender.com/gstjd1k (accessed on 14 September 2026).

**Table 1:** Structural parameters of the synthesized BiVO_4_ samples determined from XRD and Raman analysis.

Sample	Crystallite Size (D, nm)	I(040)/ I(110)	D(040) /D(121)	*a* (Å)	*b* (Å)	*c* (Å)	*β* (°)	V (Å^3^)	ν_s_(V–O) (cm^−1^)	R(V–O) (Å)
M1	62	2.32	1.30	5.1940	11.6985	5.0933	90.093	309.48	827.1	1.695
M2	57	2.54	1.24	5.1942	11.6997	5.0947	90.105	309.61	826.8	1.695
M3	59	2.42	1.26	5.1879	11.6923	5.0905	90.100	308.78	826.5	1.696
M4	65	2.70	1.27	5.1865	11.6886	5.0858	90.088	308.32	827.3	1.695
M5	65	3.36	1.28	5.1908	11.6947	5.0896	90.087	308.96	827.1	1.695
M6	60	2.98	1.24	5.1863	11.6970	5.0870	90.081	308.34	828.0	1.695
M7	60	2.58	1.26	5.1870	11.6922	5.0907	90.101	308.74	825.8	1.696
M8	61	2.85	1.28	5.1914	11.6940	5.0921	90.099	309.13	826.9	1.695
M9	62	2.79	1.29	5.1934	11.6984	5.0933	90.096	309.44	828.5	1.694

**Table 2:** Electrochemical parameters obtained from the Mott–Schottky analysis of the nine BiVO_4_ samples.

Sample	V_fb_ (V vs. SCE)	V_fb_ (V vs. RHE)	N_d_ × 10^20^ (cm^−3^)	Eg (eV)	E_CB_ ^(a)^ (V vs. RHE)	E_VB_ ^(b)^ (V vs. RHE)
M1	−0.45	0.21	1.13	2.49	0.21	2.70
M2	−0.38	0.28	1.38	2.45	0.28	2.73
M3	−0.54	0.12	1.22	2.51	0.12	2.63
M4	−0.58	0.08	1.76	2.36	0.08	2.44
M5	−0.65	0.01	2.94	2.42	0.01	2.43
M6	−0.65	0.01	1.79	2.47	0.01	2.48
M7	−0.50	0.16	1.59	2.49	0.16	2.65
M8	−0.45	0.21	1.65	2.34	0.21	2.55
M9	−0.63	0.03	1.64	2.44	0.03	2.47

^(a)^ The conduction-band edge (E_CB_) was approximated by the flat-band potential (V_fb_). Because the conduction-band minimum of an *n*-type semiconductor generally lies at a more negative potential than the flat-band potential, these values represent upper-bound estimates of (E_CB_). ^(b)^ The valence-band edge was calculated as E_VB_ = E_CB_ + E_g_; consequently, these values are also upper-bound estimates of E_VB_.

**Table 3:** Electrochemical parameters obtained from EIS fitting using the corresponding equivalent circuit model for the nine BiVO_4_ samples.

Sample	R_s_ (Ω)	R_ct_ (kΩ)	Q (μS∙s^n^/cm^2^)	n (CPE)	Ceff (μF/cm^2^)
M1	16.5	350	10.4	0.972	10.8
M2	15.6	214	11.3	0.968	11.6
M3	9.2	310	10.8	0.971	11.2
M4	19.5	117	12.2	0.953	12.5
M5	18.3	72.4	9.87	0.970	9.8
M6	18.1	110	11.1	0.975	11.2
M7	21.5	178	9.45	0.972	9.6
M8	16.1	136	10.6	0.960	10.7
M9	10.8	177	12.2	0.964	12.6

**Table 4:** Photocurrent parameters obtained from chronoamperometric measurements.

Sample	J_dark_ (μA/cm^2^)	J_peak_ (μA/cm^2^)	J_plateau_ (μA/cm^2^)
M1	0.24	1.79	1.43
M2	0.45	2.79	2.56
M3	0.51	2.05	1.68
M4	0.42	6.47	4.61
M5	0.41	9.44	6.17
M6	0.41	8.39	5.18
M7	0.38	2.92	2.22
M8	0.55	3.05	2.90
M9	0.42	2.92	2.54

**Table 5:** Photocatalytic discoloration of methylene blue (MB) under visible-light irradiation using the synthesized BiVO_4_ samples.

Sample	Adsorption (%)	Discoloration (%)	*k_app_* (min^−1^)	R^2^	t _½_ (min)
M1	11.53	28.7	0.00370 ± 0.00013	0.99862	187.3
M2	14.61	64.0	0.01139 ± 0.00053	0.98601	60.8
M3	14.79	60.8	0.01046 ± 0.00042	0.99917	66.3
M4	15.90	59.5	0.01000 ± 0.00042	0.98187	69.3
M5	17.13	86.8	0.02241 ± 0.00180	0.98265	30.9
M6	16.87	80.2	0.01804 ± 0.00087	0.98445	38.4
M7	12.83	47.8	0.00722 ± 0.00027	0.98445	96.0
M8	14.35	76.3	0.01594 ± 0.00084	0.98526	43.5
M9	12.54	73.7	0.01481 ± 0.00074	0.98678	46.8

**Table 6:** Experimental conditions employed for the synthesis of BiVO_4_ samples.

Sample	pH	Temperature (°C)	Sample	pH	Temperature (°C)	Sample	pH	Temperature (°C)
M1	0.25	160	M4	0.25	180	M7	0.25	200
M2	0.50	160	M5	0.50	180	M8	0.50	200
M3	0.75	160	M6	0.75	180	M9	0.75	200

## Data Availability

The original contributions presented in this study are included in the article and Supplementary Materials. The raw data supporting the conclusions of this article will be made available by the authors on request.

## Supplementary Materials

The following supporting information can be downloaded at https://www.mdpi.com/article/10.3390/molecules31213804/s1, Table S1: Two-way analysis of variance (ANOVA) of the 3^2^ full factorial design (factors: initial pH and hydrothermal temperature, three levels each; one observation per condition); Table S2: Correlations between the apparent rate constant (*k_app_*) and structural, optical, and electrochemical descriptors of the nine BiVO_4_ samples (*n* = 9); Table S3: Coefficients of the full quadratic response-surface model fitted to *k_app_* (10^−3^ min^−1^) in coded variables: *x*_1_ = (pH − 0.50)/0.25 and *x*_2_ = (T − 180 °C)/20 °C.
