# Supplementary material for: Structure–Property Relationships in Hydrothermally Synthesized Monoclinic Scheelite BiVO4: Role of Preferential Crystallographic Orientation in Photocatalytic Activity
Source: Molecules. 2026 Oct 22;31(21):3804. doi: 10.3390/molecules31213804 (PMC13764770; doi:10.3390/molecules31213804)
Supplement: Supplementary file 1 [file molecules-31-03804-s001.zip › molecules-4601124-supplementary.pdf]

Supplementary Materials

Structure–Property Relationships in Hydrothermally Synthesized Monoclinic Scheelite $BiVO_4$: Role of Preferential Crystallographic Orientation in Photocatalytic Activity

Freider Duran [1,*], Carlos Diaz-Uribe [1], William Vallejo [1], Ximena Vargas-Ramírez [2,3], Mario A. Millán-Franco [4,5] and Edgar Mosquera-Vargas [4,5]

[1] Grupo de Investigación en Fotoquímica y Fotobiología, Universidad del Atlántico, Puerto Colombia 081007, Colombia; [2] Grupo de Procesos Químicos y Bioquímicos Sostenibles, Universidad del Atlántico; [3] Centro de Estudios del Agua (CEA), Universidad del Atlántico; [4] Grupo de Transiciones de Fase y Materiales Funcionales, Universidad del Valle, Santiago de Cali 760042, Colombia; [5] Centro de Excelencia en Nuevos Materiales (CENM), Universidad del Valle.

* Correspondence: fgduran@mail.uniatlantico.edu.co

Contents: Table S1. Two-way ANOVA of the 3^2 factorial design. Table S2. Correlation of the apparent rate constant with structural, optical, and electrochemical descriptors. Table S3. Quadratic response-surface model for k_{app}.

Table S1. Two-way analysis of variance (ANOVA) of the 3^2 full factorial design (factors: initial pH and hydrothermal temperature, three levels each; one observation per condition).

Response	Source	SS	df	MS	*F*	*p*	% SS	Tukey *p*
k_{app} (10^{-3} min^{-1})	pH	153	2	76.29	44.40	0.002	58.1	
	Temperature	103	2	51.67	30.07	0.004	39.3	
	Residual	6.87	4	1.72	—	—	2.6	0.115
I(040)/I(110)	pH	0.2205	2	0.1102	6.95	0.050	27.5	
	Temperature	0.5171	2	0.2585	16.29	0.012	64.6	
	Residual	0.0635	4	0.0159	—	—	7.9	0.064
R_{ct} (kΩ)	pH	9149	2	4574	6.26	0.059	13.2	
	Temperature	57063	2	28531	39.07	0.002	82.5	
	Residual	2921	4	730	—	—	4.2	0.025
$J_{plateau}$ (μA cm^{-2})	pH	1.96	2	0.9794	17.53	0.010	8.9	
	Temperature	19.86	2	9.93	177.76	< 0.001	90.1	
	Residual	0.2234	4	0.0559	—	—	1.0	0.200

SS, sum of squares; MS, mean square; % SS, percentage of the total sum of squares. Because the design contains a single observation per condition, the pH × T interaction (Residual, 4 degrees of freedom) was used as the error term. Tukey *p* corresponds to Tukey's one-degree-of-freedom test for non-additivity ($p > 0.05$ indicates no significant non-additive interaction). Values of k_{app}, I(040)/I(110), R_{ct}, and $J_{plateau}$ are those reported in Tables 1, 3, 4, and 5 of the main text.

Table S2. Correlation between the apparent rate constant (k_{app}) and structural, optical, and electrochemical descriptors of the nine $BiVO_4$ samples ($n = 9$).

Descriptor	Pearson *r*	*P*	*p* (Bonferroni)	Spearman *ρ*	*p*	Leave-one-out *r* range
I(040)/I(110)	0.931	< 0.001	0.002	0.867	0.002	0.888 to 0.953
R_{ct}	-0.763	0.017	0.152	-0.750	0.020	-0.856 to -0.616
$J_{plateau}$	0.773	0.015	0.132	0.783	0.013	0.608 to 0.881
N_d	0.799	0.010	0.089	0.750	0.020	0.667 to 0.832
Dark adsorption	0.730	0.026	0.230	0.617	0.077	0.592 to 0.855
V_{fb}	-0.596	0.090	0.814	-0.546	0.128	-0.723 to -0.461
E_g	-0.384	0.308	1.000	-0.494	0.177	-0.562 to -0.261
Crystallite size D	0.257	0.504	1.000	0.093	0.812	-0.132 to 0.423
C_{eff}	-0.083	0.832	1.000	-0.017	0.966	-0.353 to 0.322

Bonferroni correction for $m = 9$ descriptors ($p_{adj} = \min(1, 9p)$). Additional robustness tests for I(040)/I(110): permutation test (20,000 permutations), $p < 0.001$; 95 % confidence interval of r by bootstrap (20,000 resamples), 0.73–0.99, and by Fisher z transformation, 0.70–0.99; partial correlation controlling for dark MB adsorption, $r = 0.860$ ($p = 0.006$); partial correlation controlling for the effective capacitance C_{eff}, $r = 0.938$ ($p = 0.001$); partial correlations were tested with $n - 3 = 6$ degrees of freedom. Removing the most active sample (M5) gives $r = 0.888$ ($p = 0.003$).

Table S3. Coefficients of the full quadratic response-surface model fitted to k_{app} (10^{-3} min^{-1}) in coded variables: $x_1 = (pH - 0.50)/0.25$ and $x_2 = (T - 180\ °C)/20\ °C$.

Term	Coefficient	Standard error	*t*	*p*
b_0 (intercept)	20.73	1.11	18.61	< 0.001
b_1 (pH)	3.73	0.61	6.12	0.009
b_2 (T)	2.07	0.61	3.39	0.043
b_{11} (pH^2)	-5.88	1.06	-5.56	0.011
b_{22} (T^2)	-6.23	1.06	-5.90	0.010
b_{12} (pH × T)	0.21	0.75	0.28	0.799

Model: $k_{app} = b_0 + b_1x_1 + b_2x_2 + b_{11}x_1^2 + b_{22}x_2^2 + b_{12}x_1x_2$. $R^2 = 0.975$; adjusted $R^2 = 0.932$; $F(5,3) = 22.9$, $p = 0.014$. Stationary point (maximum): $x_1 = 0.32$, $x_2 = 0.17$, corresponding to pH ≈ 0.58 and $T \approx 183$ °C, with a predicted k_{app} of 21.5 × 10^{-3} min^{-1}.
